# μMAPPS: a novel phasor approach to second harmonic analysis for in vitro-in vivo investigation of collagen microstructure

**DOI:** 10.1038/s41598-017-17726-y

**Published:** 2017-12-12

**Authors:** F. Radaelli, L. D’Alfonso, M. Collini, F. Mingozzi, L. Marongiu, F. Granucci, I. Zanoni, G. Chirico, L. Sironi

**Affiliations:** 1 Dipartimento di Fisica, Università degli Studi di Milano-Bicocca, Piazza della Scienza 3, 20126 Milano, Italy; 2 Dipartimento di Biotecnologie e Bioscienze, Università degli Studi di Milano-Bicocca, Piazza della Scienza 2, 20126 Milano, Italy; 3 CNR - ISASI, Institute of Applied Sciences & Intelligent Systems, Via Campi Flegrei 34, Pozzuoli, NA Italy; 4 Harvard Medical School and Division of Gastroenterology, Boston Children’s Hospital, Boston, MA USA

## Abstract

Second Harmonic Generation (SHG) is a label-free imaging method used to monitor collagen organization in tissues. Due to its sensitivity to the incident polarization, it provides microstructural information otherwise unreachable by other intensity based imaging methods. We develop and test a Microscopic Multiparametric Analysis by Phasor projection of Polarization-dependent SHG (μMAPPS) that maps the features of the collagen architecture in tissues at the micrometer scale. μMAPPS retrieves pixel-by-pixel the collagen fibrils anisotropy and orientation by operating directly on two coupled phasor spaces, avoiding direct fitting of the polarization dependent SHG signal. We apply μMAPPS to fixed tissue sections and to the study of the collagen microscopic organization in tumors *ex-vivo* and *in-vivo*. We develop a clustering algorithm to automatically group pixels with similar microstructural features. μMAPPS can perform fast analyses of tissues and opens to future applications for *in-situ* diagnosis of pathologies and diseases that could assist histo-pathological evaluation.

## Introduction

Collagen is the prominent constituent protein of tissues, providing them structural support and protection^1,2^. Since its organization changes in pathological processes^3–9^, it can be exploited as an early diagnostic marker in experimental and clinical medicine^10,11^. Collagen, myosin and microtubules in cells and tissues^12,13^ can be non-invasively imaged also *in-vivo* by Second Harmonic Generation (SHG) microscopy, a nonlinear coherent optical process where two incident photons of frequency ω are converted into a single photon of exactly twice the frequency, 2ω.

A part from the morphological information extracted from intensity-based SHG imaging^14–19^, the polarization dependence of the SHG response (P-SHG spectrum) can provide the molecular and supramolecular structure of different organs and tissues^10,20–27^. P-SHG microscopy is a promising and efficient diagnostic tool in the identification of cancer types^14,17,23^ and other pathological conditions such as ischemia, skin pathologies, fibrosis and aging^8,28–31^. In fact, P-SHG imaging, in different experimental implementations^27,32–39^ and with the support of various theoretical models of the fibrils organization, can be exploited to obtain pixel-by-pixel maps of the fibril orientation^26,40,41^, of the protein pitch angle^12,42^ and of the nonlinear susceptibility tensor components^12,15,20,43–46^. Although usually a time consuming non-linear fitting of the P-SHG spectrum of each pixel is exploited, substantial speed-up in the analysis has been recently obtained through 1D Fast Fourier Transform of the P-SHG curves^26^.

Starting from a similar Fourier Transform (FT) approach, we build here a phasor analysis of the P-SHG images that acts at a global level and identifies automatically clusters of homogenous microstructure. The phasor approach has been extensively applied to graphically analyze spectra on a variety of techniques from fluorescence lifetime imaging to MRI^47–50^, allowing to extract quantitative information on a model parameter at a pixel-by-pixel level.

We extend this global analysis by proposing a new method, named Microscopic Multiparametric Analysis by Phasor projection of P-SHG (μMAPPS), that provides micro-structural information (at pixel level) of the collagen architecture in tissues, both in terms of the mean orientation angle of the fibril (θ_F_) and of the susceptibility anisotropy parameter (γ, the ratio of off-diagonal to diagonal elements of the susceptibility tensor χ^(2)^). μMAPPS retrieves maps of both model parameters in few hundreds of ms to few seconds, by working on two coupled phasor spaces. Moreover, instead of manual and subjective clustering procedures exploited in other phasor approaches^51–53^, we develop an automated clustering algorithm, working simultaneously on the two phasor spaces, to back-project onto the original image the Regions of Interest (ROIs) characterized by similar θ_F_ and γ values. In this way we are able to automatically single out patches sharing similar micro-structural properties.

We prove that μMAPPS allows to take full advantage of the phasor approach. It provides a fast and intuitive representation of the data set, which is globally analyzed exploiting the dispersion of the data in the phasor space. In fact, differences in the microscopic behavior of the sample can be singled-out by segmenting patches of the phasor points distribution (related to the model parameters describing the system) directly in the phasor spaces and by measuring geometrical features of this distribution. Data denoising can be performed in the phasor space without affecting the spatial resolution of the original data. More important, by assuming a molecular model for the SHG response, the microstructural parameters retrieved graphically are not biased by the initial parameters values and constrains, contrary to the usual non-linear fitting procedure.

In the following a description of the μMAPPS core is presented together with its validation against simulations and *ex-vivo* experiments on mouse-tail tendons^20,54^.

The application of μMAPPS is then illustrated in the extraction of the described microscopic parameters in fixed and explanted tumor samples (biopsies). Finally, the robustness of our method has been tested in *in-vivo* experiments.

## Results

### μMAPPS Analysis Method

**Figure 1:**
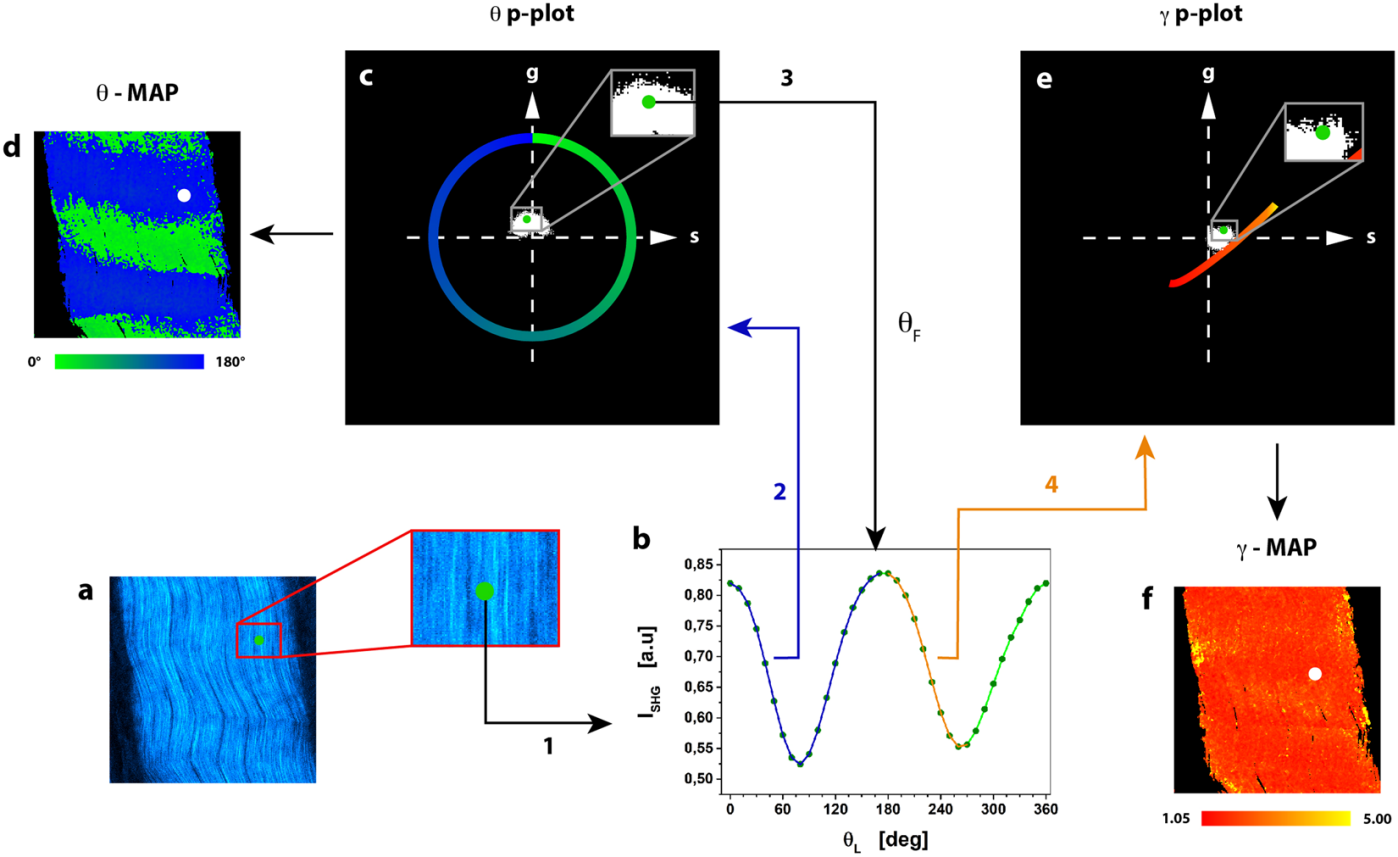
μMAPPS applied to experimental SHG image stacks from mouse-tail tendon. (**a**) Maximum intensity projection of the image stack. Image size: 225 × 225 μm^2^. (**b**) Graph of the P-SHG spectrum corresponding to the pixel highlighted by the green dot in (**a**). (**c**) θp-plot of the image stack according to Equation (1) over the [0,π) interval (blue portion of P-SHG curve in (**b**)).The green dot corresponds to the green dot in (**a**). The green-blue circle is the reference curve for γ → ∞; the color codes for the θ_F_ angles. From the θp-plot, the angle θ_F_ is retrieved by means of Equation (4) and used for the computation of the γ values. (**d**) θ-map obtained pixel by pixel from the θp-plot (Equation (4)). (**e**) γp-plot of the image stack according to Equation (2) over the range [θ_F_, θ_F_ + π/2) (orange portion of P-SHG curve in (**b**).The green dot corresponds to the green dot in (**a**). The red-yellow curve is the reference for the γp-plot obtained for 0 ≤ γ ≤ 10 and Δθ = 10° assuming, as an example, the specific value θ_F_ = 0°. (**f**) γ-map obtained pixel by pixel from the γp-plot by means of Equation (5).

**Figure 2:**
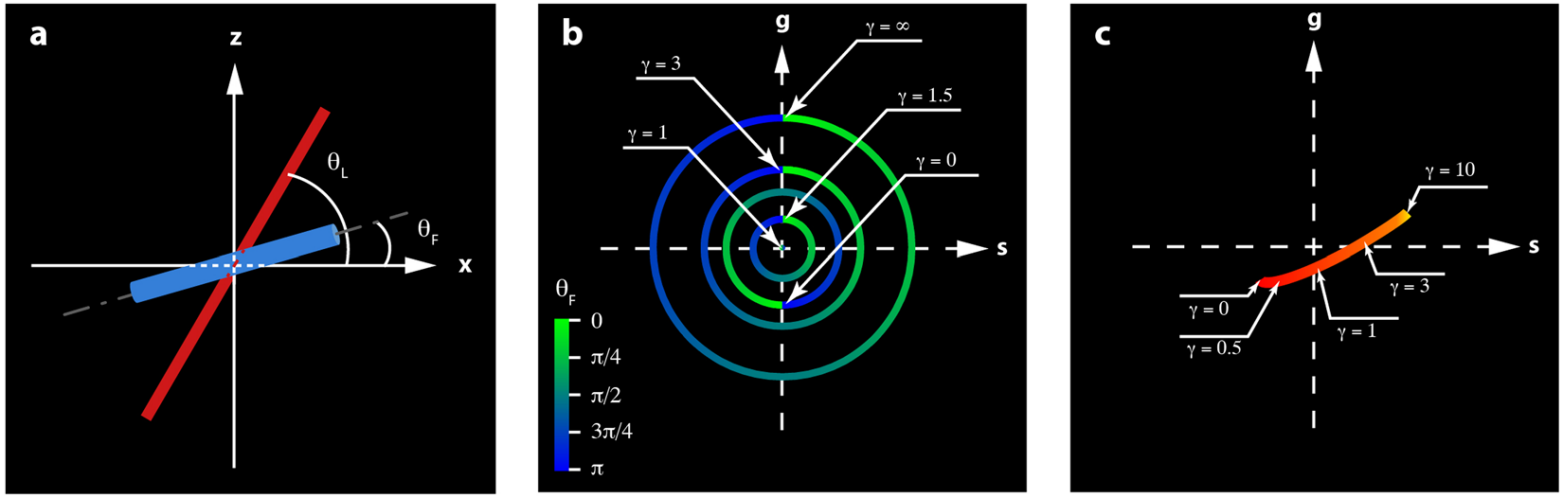
Reference system for the P-SHG measurements. (**a**) Coordinate system scheme: the laser is incident along the y-direction, while the collagen fibrils lie in the xz-plane. θ_L_ and θ_F_ are, respectively, the laser excitation polarization angle and the fibril orientation angle with respect the x-axis direction. (**b**) Reference curves for the θp-plot, obtained from Equations (1) and (3) for different values of γ and fixed value of θ_F_, as a function of θ_L_ in the range [0, π). For γ > 1 the radius of the circle increases as γ increases; for 0 ≤ γ < 1 the radius increases as γ decreases and it is simultaneously π-tilted. For γ = 1, the reference curve degenerates to a point in the origin. For 0 < γ < 1 and 1 < γ < 2.1375 (numerically obtained) the reference circles may be superimposed, although π shifted. The angle in the θp-plot is double the θ_F_ value. (**c**) Reference curve for the γp-plot. The mapping of the pixel on the γp-plot depends only on γ and falls, apart from data non-idealities, on the reference curve. For the simulations, a data sampling value Δθ = 1° was used.

The μMAPPS projection method starts from the acquisition of a stack of images as a function of the laser polarization θ_L_. The maximum intensity projection of the stack provides the overall disposition of the collagen in the sample (Fig. 1a). The P-SHG spectrum (SHG as a function of θ_L_, Fig. 1b) of each pixel is projected onto a first complex plane (called θp-plot, Fig. 1c), via the first harmonic normalized Discrete Fourier Transform (DFT) applied to the 0 ≤ θ_L_ < π range. Each pixel is transposed into a point, whose coordinates are the real and the imaginary parts of the signal DFT (see also Supplementary Notes 1–2 and Supplementary Figs. 1–3). The points in the θp-plot spread on a cloud around the origin (Fig. 1c) and their absolute position depends on both the collagen fibrils angle θ_F_ (Fig. 2a) and the microscopic order parameter. On the contrary, the angular position of these points in the θp-plot depends only on the collagen fibrils angle θ_F_. A second phasor plot (called γp-plot, Fig. 1e), is then computed by DFT of the P-SHG in the domain θ_F_ ≤  θ_L_ < θ_F_ + π/2 (Fig. 1b). The pixel projection in the γp-plot is related to the microscopic order parameter.

By mapping the P-SHG spectrum onto the two phasor plots we are therefore able to retrieve both the fibril orientation and the local order parameter pixel by pixel and build a θ-map (Fig. 1d) and γ-map (Fig. 1f) of the sample from a stack of images. We have verified that μMAPPS (Equations (4–5)) provides results in agreement with the direct fitting of the P-SHG spectra to Equation (3).

More in details, the coordinates (g_θ_, s_θ_) of a point in the θp-plot are the first DFT of the normalized P-SHG curve, ${\{I({\theta }_{L}^{n})\}}_{n=\mathrm{0..}N-1}$, acquired as a function of θ_L_ (0 ≤ θ_L_ < 3π/2) with resolution Δθ. We define the frequency ${K}_{\theta }=2\pi {(N{\rm{\Delta }}\theta )}^{-1}$ and compute these coordinates as (N = π/Δθ):

1 $${g}_{\theta }=\frac{\sum _{n=0}^{N-1}I({\theta }_{L}^{n})\,\cos \,({\theta }_{L}^{n}{K}_{\theta })}{\sum _{n=0}^{N-1}I({\theta }_{L}^{n})};\,{s}_{\theta }=\frac{\sum _{n=0}^{N-1}I({\theta }_{L}^{n})\sin ({\theta }_{L}^{n}{K}_{\theta })}{\sum _{n=0}^{N-1}I({\theta }_{L}^{n})}$$

Each pixel is projected by Equation (1) onto the θp-plot and from its angular position it is possible to estimate the fibril orientation θ_F_ (Fig. 1c). This value corresponds to orientation of the fibril with respect to the polarization axis.

The anisotropy of the SHG susceptibility is measured by building a second phasor plot, called γp-plot, that depends on the estimated value θ_F_ and whose coordinates are defined as $({K}_{\gamma }=2\pi {[N({\rm{\Delta }}\theta +{\theta }_{F})]}^{-1})$:

2 $${g}_{\gamma }=\frac{\sum _{n=0}^{N/2-1}I({\theta }_{L}^{n}+{\theta }_{F})\cos (({\theta }_{L}^{n}+{\theta }_{F}){K}_{\gamma })}{\sum _{n=0}^{N-1}I({\theta }_{L}^{n}+{\theta }_{F})}\,;\,{s}_{\gamma }=\frac{\sum _{n=0}^{N/2-1}I({\theta }_{L}^{n}+{\theta }_{F})\sin (({\theta }_{L}^{n}+{\theta }_{F}){K}_{\gamma })}{\sum _{n=0}^{N-1}I({\theta }_{L}^{n}+{\theta }_{F})}$$

In this case, we compute the DFT in the θ_F_ ≤ θ_L_ < θ_F_ + π/2 range. In this way, we are maximally sensitive to the shape of the P-SHG spectrum (see Supplementary Fig. 2) with minimal influence from θ_F_. The position of the projection (g_γ_, s_γ_) in the γp-plot (Fig. 1e) is a measure of the P-SHG shape. The latter can be related, through a suitable model, to the local disorder of the fibrils.

Only P-SHG spectra above a threshold (T_NL_) are analyzed in order to discard the background contribution.

By exploiting the graphical analysis in the two linked phasor plots, we can map the P-SHG spectra of each pixel to the microscopic features of the sample. In order to quantify the degree of collagen disorder in the tissue, we need to choose a microscopic model for the SHG response and build in the θp-plot and γp-plot two reference curves. We adopt here a widespread theoretical model^15,20,43^ to describe the relation among the SHG signal, $I({\theta }_{L}^{n})$, the relative angle ${\theta }_{L}^{0}-{\theta }_{F}$ between the laser polarization and the collagen fibrils orientations, and the anisotropy of the second order susceptibility ${\underline{\underline{\chi }}}^{(2)}$ tensor, $\gamma ={\chi }_{zzz}^{(2)}/{\chi }_{zxx}^{(2)}$

3 $$I({\theta }_{L}^{n})=k\{{\sin }^{2}[2({\theta }_{L}^{n}-{\theta }_{F})]+{[{\sin }^{2}({\theta }_{L}^{n}-{\theta }_{F})+\gamma {\cos }^{2}({\theta }_{L}^{n}-{\theta }_{F})]}^{2}\}$$

The parameter γ > 0 (ranging from γ ≅ 0.5 for myosin^27,40^ to γ ≅ 1.5–2 for collagen^54^) describes the molecular anisotropy and the scale factor k includes the absolute intensity of the SHG signal as affected by the setup parameters. Equation (3) is valid under the assumption of cylindrical (C_∞_) or hexagonal (C_6_) plus Kleinman symmetries (see also Supplementary Note 1).

We build therefore reference curves in the θp-plot and the γp-plot by applying the Equations (1–2) to the microscopic model described by Equation (3) as a function of γ and θ_F_. A closed circular curve (green-blue color coded curve in Fig. 2b) is obtained in the θp-plot by varying the fibril angle θ_F_ in the range [0,π], while keeping γ constant. γ affects the θp-plot reference curve by changing its radius (Fig. 2b). For γ > 1 the circle radius increases with γ. For 0 ≤ γ < 1 the radius increases as γ decreases and it is simultaneously π-tilted. For γ = 1 the circle collapses at the phasor plot origin. Therefore, the angular position of the projection of the P-SHG spectrum on the θp-plot determines univocally the fibril angle θ_F_ according to the following algorithm:

4 $$\cos (2{\theta }_{F})=\frac{{g}_{\theta }}{\sqrt{{g}_{\theta }^{2}+{s}_{\theta }^{2}}}$$

θ_F_ is half the value of the angle that the vector $({g}_{\theta },{s}_{\theta })$, pointing from the center of the θp-plot to the pixel projection, makes with the phasor plot imaginary axis (see Supplementary Fig. 4). The reference curve of the γp-plot (Fig. 2c) is then built by DFT transforming (Equation (2)) the P-SHG spectrum (Equation (3)). We obtain an open reference curve (Fig. 2c, red-yellow color code) which is, unless for data sampling effects, independent of the θ_F_ value. The parameter γ is measured by retrieving the minimum Euclidean distance projection onto the Reference Curve (RC, see Supplementary Fig. 4) defined as:

5 $$\{\begin{array}{ccc}{d}_{e-RC} & = & \sqrt{{({g}_{e}-{g}_{RC})}^{2}+{({s}_{e}-{s}_{RC})}^{2}}\\ {\gamma }_{e} & = & {{\gamma }_{RC}|}_{{\rm{\min }}({d}_{e-RC})}\end{array}$$

In Equation (5) the minimum is searched over the points of the γp-plot reference curve computed with the θ_F_ value measured on the first phasor plot (Equation (4)) and with the experimental sampling angle Δθ.

The algorithm for the γ-map retrieval (Equation (5)) was modified in order to take into account the onset, in tumor samples, of a non-negligible background value (y_0_ term added to Equation (3)), probably due to an intra-pixel fibrils distribution. We studied the mutual dependence between γ and y_0_ and modified the procedure to retrieve the γ values by minimizing the angle between the (g_γ_, s_γ_) vector and all vectors spanning the reference curve (Supplementary Note 3 and Supplementary Fig. 5).

It should be noted that our graphical method, by acting on a double phasor space, goes beyond the measure of the phase of the first Fourier component of the P-SHG spectrum as done in ref.^26^, which is equivalent to our Equation 4. Our double phasor space approach allows us to quantify both θ_F_ and γ parameters, avoiding the algebraic solution proposed in ref.^26^ to determine γ, and exploiting the denoising opportunities of the phasor approach.

### Validation through numerical simulations

The μMAPPS core has been thoroughly validated against numerical simulations. μMAPPS is able to tackle with nonideal experimental conditions such as the laser polarization sampling Δθ and the noise (see Supplementary Fig. 6). For Δθ → 0° the γp-plot reference curve is strictly independent of θ_F_. Non ideal data sampling (for example Δθ = 10° as in our experimental condition) results in the shift of the points from the ideal reference curve in the γp-plot. This is due to the fact that we compute the point coordinates (g_γ_, s_γ_) on the P-SHG spectrum shifted by an angle that is the nearest sampled θ_F_ value estimated from the θp-plot. Moreover, two P-SHG spectra characterized by θ_F_ values differing by a multiple integer of Δθ (and γ_1_ = γ_2_), have similar projections on the γp-plot reference curve. In fact, exactly the same (θ_F_ ≤ θ_L_ < θ_F_ + π/2) portion of identical P-SHG spectra are DFT analyzed in this case.

By working in the phasor plots, we are able to efficiently tackle with the experimental noise. P-SHG Gaussian noise scatters the projections in both phasor plots. This effect is enhanced if combined to a distribution of γ and θ_F_ over the whole image stack (see Supplementary Fig. 7) or if a Gaussian distribution of fibrils angles within each pixel is considered (see Supplementary Fig. 8). A denoising filter was applied directly in the phasor space in order to reduce the dispersion of projected spectra without affecting the P-SHG intensity images^55^. In our case, a median filter with a 3 × 3 pixels mask was directly and independently applied to the *s* and *g* coordinates (Supplementary Note 4 and Supplementary Fig. 9). In this way, it is possible to reduce the dispersion of the projections in the θp-plot and γp-plot by 4 ± 0.6 and 3.4 ± 0.3 times, respectively.

Finally, μMAPPS allows us to solve efficiently also an intrinsic degeneracy of the model (Equation (3)). As shown in Supplementary Fig. 10, P-SHG spectra with θ_1_ = θ_F_ and 1 ≤ γ_1_ < 2.1375 (numerically determined) and θ_2_ = θ_F_ + π/2 and 0 < γ_2_ < 1 may fall on the same position on the γp-plot reference curve. In these cases, the experimental P-SHG spectrum is then directly compared to the model (Equation (3)) only for the couples θ_F_ = θ_1_, γ = γ_1_ (within the range [θ_1_, θ_1_ + π/2]) and θ_F_ = θ_2_, γ = γ_2_ (within the range [θ_2_,θ_2_ + π/2]). The solution with the minimum chi-squared value from the experimental P-SHG is selected. This procedure can be avoided if the type of collagen (e.g. Collagen I is characterized by values of γ > 1) is a priori known.

### Data clustering

For tissue analysis, it is essential to identify pixels characterized by similar microstructural parameters. Instead of manually (and somehow arbitrary) selecting different areas in the phasor plot and map the corresponding pixels back onto the morphological image, we develop here a data clustering approach (please refer to Supplementary Note 5).

Our clustering algorithm works pixel-by-pixel in the θ_F_ − γ space. It first identifies the putative clusters’ centers according to a maximum density approach^56^ over both the θ_F_ and γ parameters. Each element which is not a putative center is assigned, by an iterative procedure, to the cluster for which the element-center distance is the lowest. The validation of the clustering algorithm on P-SHG simulated noisy spectra (Supplementary Fig. 11) shows that the results are independent from the pixels location within the acquired image stack and the pixel average SHG intensity. Pixels characterized by parameters θ_F_ and γ within the cutoffs are then superimposed on the morphological image with a color code.

### Validation through measurements

**Figure 3:**
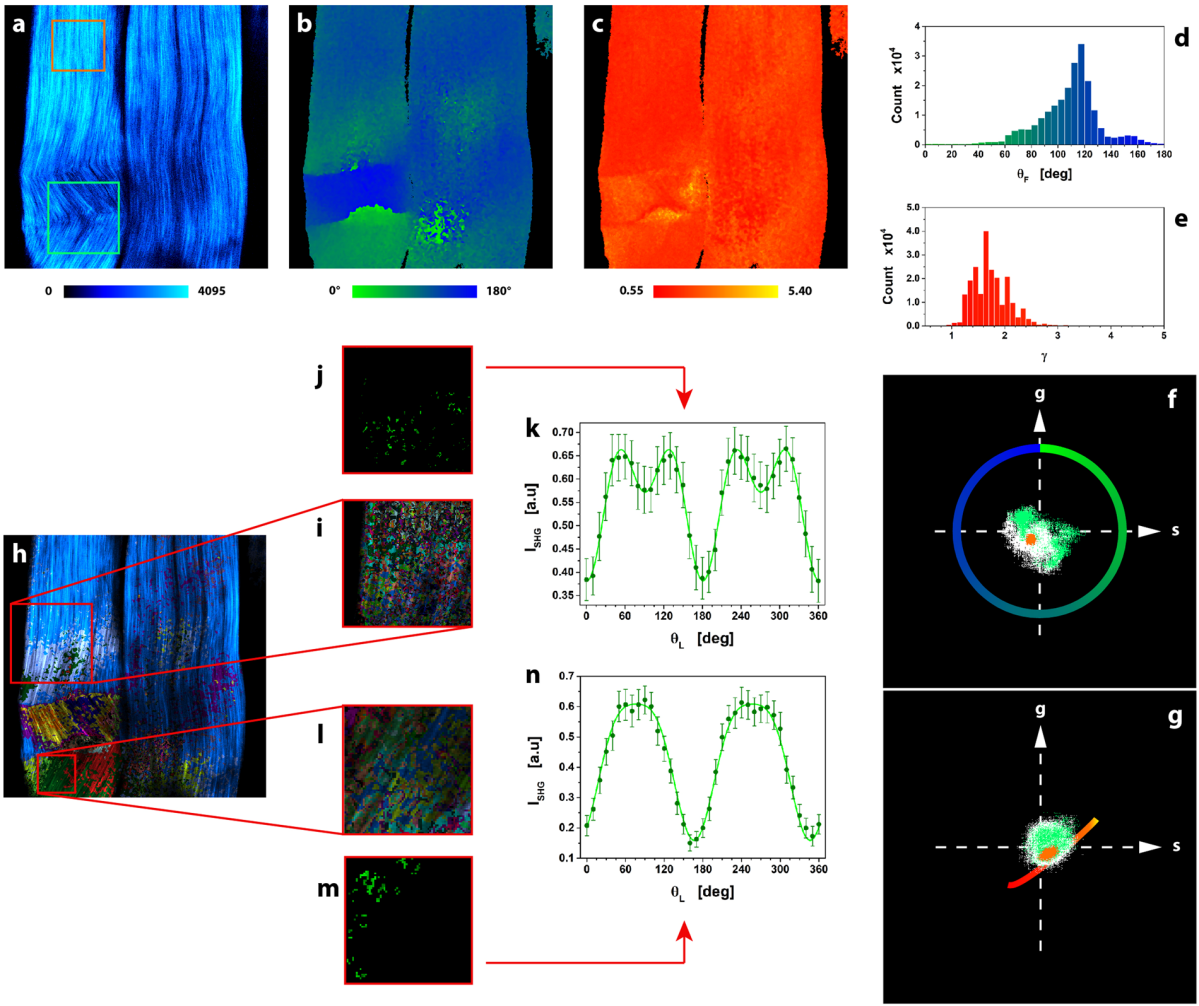
μMAPPS analysis of mouse-tail tendon. (**a**) Maximum intensity projection of the image stack (Δθ = 10°). Image size: 225 × 225 μm^2^. (**b** and **c**) represent respectively the pixel by pixel θ- and γ- maps, where the color codes are shown in the legends. (**d** and **e**) are the counts histograms extracted from the θ- and γ- maps. (**f** and **g**) show the θp-plot and γp-plot respectively, from which the θ- and γ-maps are derived according to Equations (4) and (5). The small compact cloud of points in the θ- and γp-plots (orange) corresponds to the uniform area highlighted in (**a**) with an orange box, while a non-homogeneous microscopic behavior (green box in **a**) causes points scattering (green) in both phasor plots. (**h**) Result of clustering procedure, highlighting the different microscopic behavior in the tissue. 23 clusters were obtained with the following cutoff values: θ_C_ = 30° and γ_C_ = 1. ET = 1%. The threshold noise level was T_NL_ = 250. Each monochromatic LUT encodes for a cluster with an intensity scaling as the pixel integrated P-SHG signal. (**i** and **l**) show two ROIs (i: 154 × 154 pixels; l: 69 × 69 pixels) selected from (**h**) and analyzed with tighter cutoff values (θ_C_ = 2°, γ_C_ = 0.1 and ET = 1%) to select clusters with more similar microscopic behavior. One cluster, selected from each ROI in (**i**) and (**l**), was reported in (**j** and **m**). The average P-SHG spectrum of the two selected clusters (reported in (**k)** and (**n**)) were fit to the model Equation (3). The best fit parameters θ_F_ = 91.0° ± 0.4° and γ = 1.43 ± 0.01 (cluster j), θ_F_ = 76.5° ± 0.6° and γ = 2.09 ± 0.05 (cluster m), are in excellent agreement with the μMAPPS estimates θ_F_ = 92.2° ± 0.6° and γ = 1.45 ± 0.05 (cluster j), θ_F_ = 77.9° ± 0.6° and γ = 2.16 ± 0.05 (cluster m).

μMAPPS was validated at first on the intense SHG signals produced by the highly organized and regular fibers of the mouse-tail tendon^20,32,54^. Figure 3a reports the maximum intensity projection images for a native mouse tendon. The θ-map and γ-map (Figs. 3b,c) were obtained by applying μMAPPS to the θ- and γp-plots (Figs. 3f,g). The γ values were obtained by projecting the P-SHG spectra onto the reference curve of the γp-plot (calculated with Δθ = 10° and θ_F_ retrieved from the first phasor plot) as in Equation (5).

As highlighted in Fig. 3, qualitative information on the microstructure heterogeneity can be obtained also without assuming any theoretical model (RC): uniform regions (orange box in Fig. 3a) correspond to a small compact cloud of points (orange in Figs. 3f,g) in the θ- and γp-plots, while microscopic non-homogeneity (green box in Fig. 3a) results in clouds of scattered points (green in Figs. 3f,g). A mean fibrils angle <θ_F_> = 116° ± 5° is obtained for the more uniform region in Fig. 3a (as from Gaussian fit of the θ_F_ distribution in the orange box), with low anisotropy values (γ < 2). Regions that correspond in the morphological image to crimped features (Fig. 3a, green box) have a double distribution of the fibril angles, at <θ_F_> = 72° ± 4° and <θ_F_> = 154° ± 4°, and high anisotropy values (γ > 2). Over the whole image in Fig. 3 the distribution of the anisotropy value is wide with <γ> = 1.7 ± 0.6 (as from the Gaussian fit of the distribution in Fig. 3e) in agreement with the literature^20,32,54^ and the distribution of the fibrils angle is peaked at θ_F_ ≅ 116° with two secondary components at θ_F_ ≅ 80° and at θ_F_ ≅ 155°. A more quantitative analysis of the spatial in-homogeneity of the tissue can be obtained starting from the application of our clustering algorithm to the two coupled phasor spaces. Figure 3h shows 23 clusters encoded with different monochromatic linear LUTs (coding for the integrated SHG intensity of the pixel): the blue color highlights the main cluster, containing the 57% of the total elements (122˙998) and corresponding to a uniform regions with <θ_F_> = 113° ± 7° and <γ> = 1.7 ± 0.2.

To further validate the μMAPPS algorithm, we compared our results with those from fitting-based procedures. The fit of the P-SHG spectra, obtained from clusters retrieved with tight cutoff values on two ROIs (θ_C_ = 2° and γ_C_ = 0.1, Figs. 3i,j and l,m), are reported as an example in Figs. 3k and n, showing and excellent agreement between the two methods. μMAPPS was then also applied to samples of mouse-tail tendon denatured via collagenase digestion, retrieving again results in agreement with the literature^57^ (see Supplementary Note 6 and Supplementary Fig. 12).

### μMAPPS analysis of histology tumor sections

**Figure 4:**
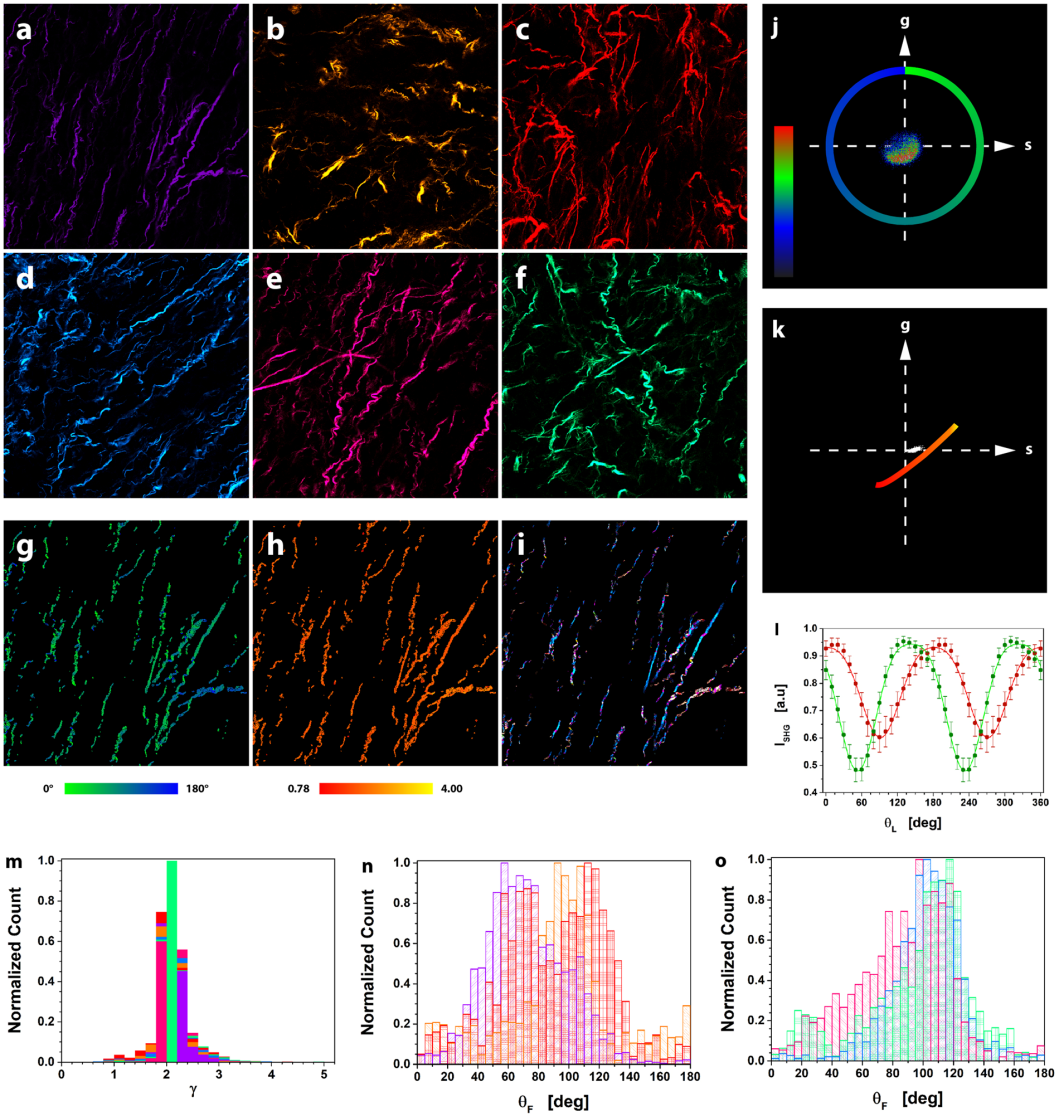
μMAPPS analysis of tumor biopsies. (**a**–**f**) Maximum intensity projection of the image stack related to six different FOVs (Δθ = 10°). Panels (a–c) are related to three different FOVs of a tumor section, while panels (**d**–**f**) show three FOVs from a second tumor section. Image size: 283 × 283 μm^2^. (**g** and **h**) show the θ-map and the γ-map of the FOV in (**a**). (**i**) Result of the clustering procedure on the FOV in (**a**). 10 clusters were obtained with cutoff values θ_C_ = 5° and γ_C_ = 0.2; ET = 2%. Each monochromatic LUT encodes for a cluster with an intensity scaling as the pixel integrated P-SHG signal. (**j** and **k**) represent, respectively, the θp-plot and γp-plot of the FOV in (**a**). The color scale encodes for the counts per phasor plot pixel. (**l**) The average P-SHG spectra related to two clusters (cutoff values θ_C_ = 2° and γ_C_ = 0.1) were fit to the model Equation (3), with the addition of the background y_0_ term (lines). The best fit parameters are: θ_F_ = 143.2° ± 0.4°, γ = 2.11 ± 0.03, y_0_ = 0.35 ± 0.01, k = 0.13 ± 0.01 (green) and θ_F_ = 1.5° ± 0.6°, γ = 2.21 ± 0.06, y_0_ = 0.52 ± 0.01, k = 0.08 ± 0.01 (red). The μMAPPS results are: θ_F_ = 143.2° ± 0.3°, γ = 2.18 ± 0.03, (green) and θ_F_ = 3.0° ± 0.6°, γ = 2.19 ± 0.03 (red). The threshold noise level for all analyses was T_NL_ = 200. (**m**) shows the γ counts histograms related to the six FOVs in (**a**–**f**), with the same color code. (**n** and **o**) show the θ-histograms for the FOVs in (**a**–**c**) and (**d**–**f**), respectively. Each histogram is shown with the same color code of the related image.

Alterations in the ExtraCellular Matrix (ECM), whose prominent constituent is collagen, are correlated to tumor development: the increased collagen deposition in some tissues facilitates tumor initiation and progression^3,4,10,14,58,59^. Therefore, we evaluated the efficacy of μMAPPS to characterize the ECM of fixed sections of CT26 derived tumor from mice (5 μm thickness). The average SHG signal from collagen reveals thin wavy fibers in the ECM of tumor samples explanted 7 days after inoculation, as shown in Figs. 4a–f for six fields of view (FOVs) from sections obtained from two different samples. Figures 4g,h show the θ-map and γ-map of the FOV in Fig. 4a, obtained by analyzing data of the θp-plot and the γp- plot (Figs. 4j,k) through Equations (4–5). The γ and θ counts histograms related to the six FOVs are reported in Figs. 4m–o with the same color-code as in Figs. 4a–f. The retrieved anisotropy parameter spans the range [0.78–4] within each FOV (Fig. 4m), whose average γ values lie within [2.06 ± 0.18–2.10 ± 0.20], and the overall mean value is <γ> = 2.08 ± 0.04. The fibrils angle obtained within each FOV is characterized by quite large distributions with widths in the [8°–22°] range. Again, microscopic parameters can then be grouped and highlighted in the original image by using our cluster algorithm (Fig. 4i). In our analysis we found a mean of 8 ± 2 clusters (cutoff values θ_C_ = 5° and γ_C_ = 0.2; ET, minimum number of elements in a cluster with respect to the total number of analyzed pixels, =2%).

In the tumor samples the presence of non-negligible background values made us modify the γ-map retrieval algorithm (Supplementary Fig. 5). Therefore, we validated again μMAPPS against fitting-based procedures: Fig. 4l reports an example of the fit of the P-SHG spectra extracted from two different clusters (θ_C_ = 2° and γ_C_ = 0.1), leading to parameters in excellent agreement with μMAPPS results. This successful validation not only demonstrates that μMAPPS is able to obtain microscopic parameters in agreement with fitting-based algorithms, but also that it is a versatile method that can afford deviations from the theoretical model described by Equation (3).

### μMAPPS analysis of tumor biopsies

**Figure 5:**
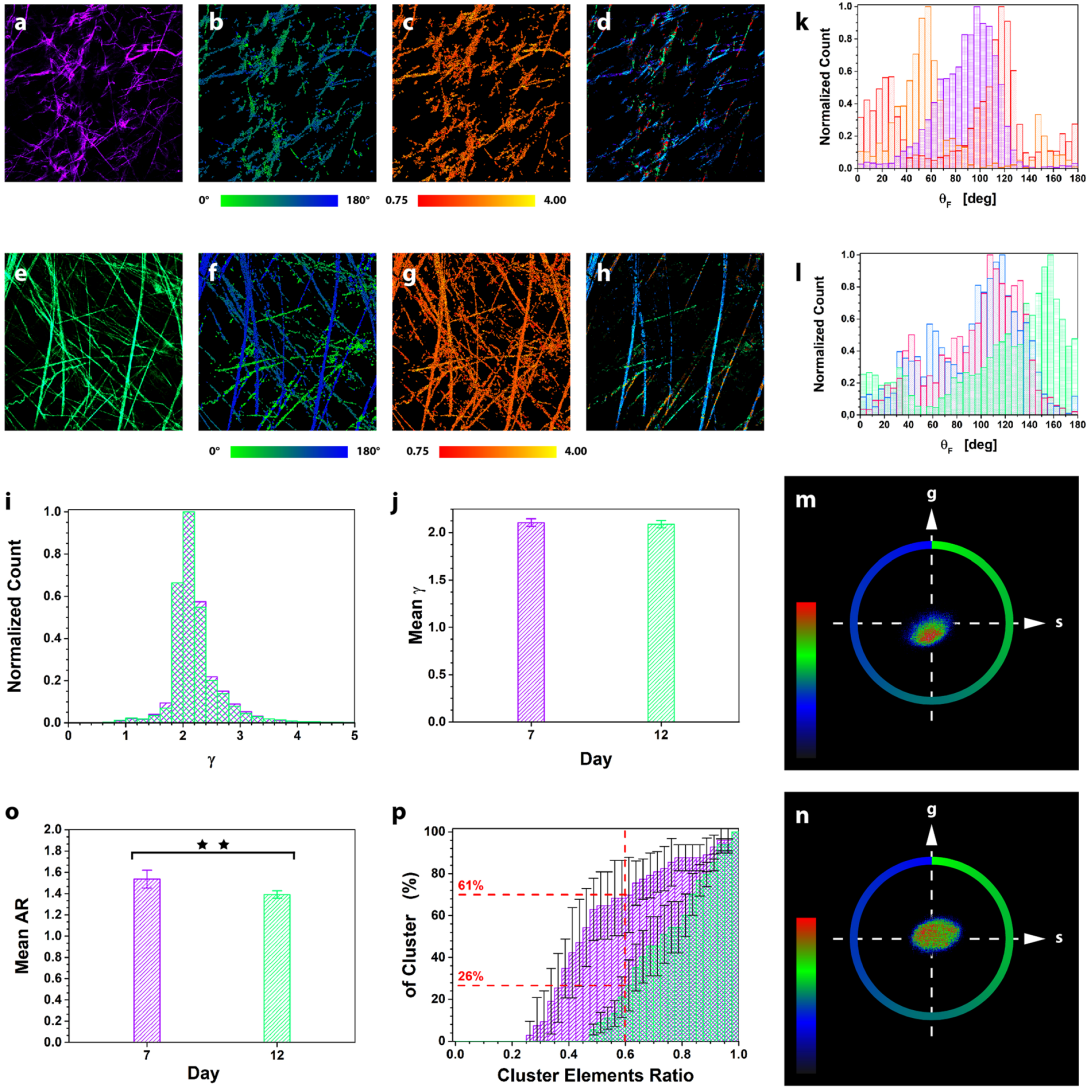
μMAPPS analysis of tumor biopsies. (**a** and **e**) show, respectively, the maximum intensity projection of the SHG image stack for a tumor biopsy at day 7 and at day 12 out of a total 30 FOVs (3 biopsies) analyzed. Image size: 225 × 225 μm^2^. (**b**,**c**) θ- and γ-maps of the image in (**a**). (**d**) Result of the clustering of the tumor biopsy at day 7 in (**a**) with cutoff parameters: θ_C_ = 30°, γ_C_ = 1, ET = 10%, T_NL_ = 200. The P-SHG intensity of the pixels of a cluster is encoded by a monochromatic LUT. (**f**,**g**) θ- and γ-maps of the image in (**e**). (**h**) Clustering procedure (θ_C_ = 30°, γ_C_ = 1, ET = 10%, T_NL_ = 200) for the tumor biopsy at day 12 in (**e**). (**i**) The γ counts histograms related to all our experiments performed at day 7 (purple) and day 12 (green) are superimposed. (**j**) shows the mean γ value for all the samples at day 7 (purple), < γ >  = 2.11 ± 0.04, and at day 12 (green), < γ >  = 2.09 ± 0.04. (**k** and **l**) report the θ-count histograms for the biopsies excised at day 7 and day 12, respectively. Each color codes for a different FOV selected in a tumor biopsy at day 7 and at day 12. (**m** and **n**) show the two θp-plots for the FOVs reported respectively in (**a** and **e**). The color scale encodes for the counts per phasor plot pixel. (**o**) Ratio (AR) between the major and minor axis of the ellipse enclosing the points for tumors at day 7 (purple) and 12 (green). Note that two asterisks (**) have the significant meaning 0.001 < p < 0.01, based on two-tailed Student’s t-test. (**p**) Cumulative distribution of the number of clusters (reported as percentage) as a function of the ratio between the number of elements in each retrieved cluster and the most populated cluster (CER).

Here we report our preliminary results on tumor ECM studies in biopsies excised at day 7 and 12 after B16 melanoma cells implantation in the left flank of mice. We found that the tumor structure is heterogeneous throughout the sample, depending on the depth from the tumor surface and the size of the selected FOV. At day 12 the collagen fibers appear straight below the tumor cap, whereas they are curly at the surface. At day 7 fibers are wavier, shorter and less dense with respect to day 12. Due to this heterogeneous environment, we chose to perform our experiments at a fixed z position range below the tumor surface (10–15 μm). Two exemplary images of a tumor at day 7 and 12, out of 30 FOVs from 3 biopsies, are shown in Figs. 5a and e, respectively. By applying μMAPPS, we extracted the θ_F_ and γ maps for the tumor at day 7 (Figs. 5b,c) and at day 12 (Figs. 5f,g). The γ distributions (Fig. 5i) computed over all the experiments at day 7 (purple) and the day 12 (green) show no change. In each FOV, γ is variable in the range [0.9–4], while the mean values obtained by averaging over the different FOVs, are: <γ> = 2.11 ± 0.04 (day 7) and <γ> = 2.09 ± 0.04 (day 12) (Fig. 5j). The width of the fibrils angular distribution within each FOV lies in the range [8°–19°] at day 7 (Fig. 5k), while it is larger at day 12 (Fig. 5l), variable within [13°–26°], as obtained by a multi-Gaussian fit.

Differences in the microscopic organization of the investigated samples can be also extracted by a global analysis of the shape of the cloud of points within the θp-plot (Figs. 5m,n). Although the distribution of the γ values is not changing between day 7 and day12 (Fig. 5i), different shapes of the θp-plots for the two time-points are retrieved and characterized by measuring (Fig. 5o) the ratio (AR) between the major and minor axis of the ellipse enclosing the points in the phasor plot. At day 12, <AR> = 1.39 ± 0.03, while at day 7 <AR> = 1.53 ± 0.08, indicating a more isotropic fibrils angular distribution within each FOV at day 12. The phasor analysis is therefore able to obtain results in agreement with Figs. 5k,l, and can help in highlighting differences between the samples faster than histogram construction and peak fitting procedures.

We further characterize the tumor biopsies by applying the clustering algorithm. First, we assumed large clustering parameters (θ_C_ = 30° and γ_C_ = 1 ET = 10%) in order to evaluate the mean Cluster Elements Ratio (CER, ratio between the number of elements in each retrieved cluster and in the most populated cluster) and to single out the presence of a main cluster. At day 12 the <CER> is 0.54 ± 0.14, while < CER > = 0.14 ± 0.14 at day 7, indicating the presence of a major cluster at day 7. Clusters obtained with these large cutoff parameters are reported in Figs. 5d and h.

Small scale heterogeneity in the tissue can be singled out by looking for clusters with few elements. To this purpose, we clustered the data set with more tight conditions (θ_C_ = 5° and γ_C_ = 0.2; ET = 2%), obtaining the results summarized by the cumulative distributions of Fig. 5p. At day 7 the tumors are characterized by a higher number of clusters with small CER with respect to day 12. In fact, the 60% of clusters at day 7 (purple) have a CER value below 0.5, while this percentage is reduced to 26% at day 12 (green). These results suggest the presence of a higher number of clusters with few elements at day 7 with respect to day 12, characterized instead by clusters with a more uniform population.

### μMAPPS *in-vivo* analysis of tumors

**Figure 6:**
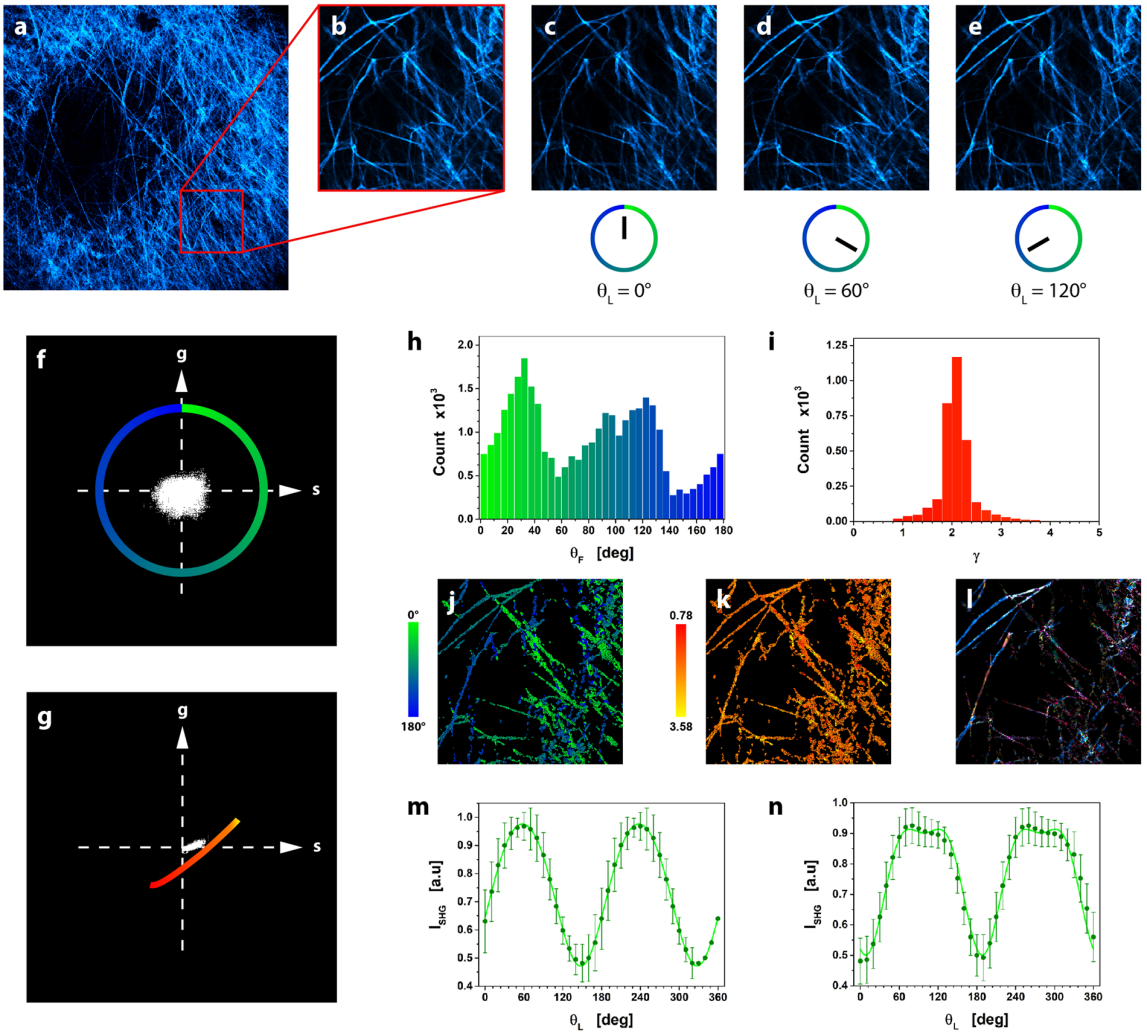
μMAPPS analysis of tumor *in-vivo*. (**a**) A stack of images (564 × 564 μm^2^) comprising 30 z-planes acquired at 1 μm z spacing, from 10 to 40 μm below the tumor ECM surface, have been z-projected in the image for θ_L_ = π. (**b**) ROI for *in-vivo* μMAPPS analysis (225 × 225 μm^2^). (**c**–**e**) Three images (θ_L_ = 0°, θ_L_ = 60° and θ_L_ = 120°) corrected for motion artifacts (see methods). (**f** and **g**) are the θp-plot and γp-plot computed on ROI in (**b**). (**h** and **i**) report the corresponding counts histograms. (**j** and **k**) represent the θ and γ maps obtained from (**f** and **g**), respectively. (**l**) Output of the clustering algorithm on the ROI in (**b**) with θ_C_ = 5°, γ_c_ = 0.2, ET = 2%, T_NL_ = 300. The P-SHG intensity of the pixels of a cluster is encoded by a monochromatic LUT. (**m** and **n**) Average P-SHG spectra for two clusters (obtained with θ_C_ = 2°, γ_c_ = 0.1 and T_NL_ = 200), fit to the model Equation (3) with the addition of the background y_0_ term (lines). The best-fit parameters are: γ = 2.57 ± 0.03, θ_F_ = 57.6° ± 0.2°, y_0_ = 0.38 ± 0.01, k = 0.09 ± 0.01 (cluster m); γ = 1.83 ± 0.04, θ_F_ = 98.3° ± 0.7°, y_0_ = 0.33 ± 0.02, k = 0.17 ± 0.01 (cluster n), in agreement with the μMAPPS values that are γ = 2.39 ± 0.03, θ_F_ = 56.4° ± 0.3° (cluster m) and γ = 1.90 ± 0.04, θ_F_ = 95.2° ± 1.1° (cluster n).

In order to demonstrate that our method is robust also against possible artifacts affecting intravital experiments, μMAPPS was also applied to study the micro-structure of ECM in developing tumors directly in living-anesthetized animals. Figure 6a reports the maximum intensity z-projection (10 to 40 μm below the tumor surface) of a SHG image stack (564 × 564 μm^2^, θ_L_ = 2π) in the tumor ECM *in-vivo*. A ROI (Fig. 6b) was then selected for μMAPPS analysis (see Figs. 6c–e for an example of motion-corrected images). The θ and γ maps (Figs. 6j,k) and histograms (Figs. 6h,i) were obtained from the corresponding θp-plot (Fig. 6f) and γp-plot (Fig. 6g) by taking into account also the background effect (finite y_0_ value). The Gaussian fit of the anisotropy distribution (Fig. 6i) in the selected FOV gave <γ > = 2.07 ± 0.19, in agreement with biopsies, indicating that both experimental conditions can be used for collagen characterization. Moreover, clusters retrieved with the cutoff parameters θ_C_ = 5° and γ_c_ = 0.2 (ET = 2%), are shown in Fig. 6l. A close agreement between fitting and μMAPPS results demonstrates the validity of our method in the evaluation of microstructural parameters also *in-vivo* (Figs. 6m,n).

## Discussion

μMAPPS is based on the Fourier projection of the P-SHG stacks of images onto two concatenated phasor plots from which, by a simple geometrical construction and the assumption of a molecular model, we can extract distributions of the collagen fibrils angle, θ_F_, and of the anisotropy parameter, γ. The robustness of μMAPPS in retrieving θ_F_ and γ was numerically tested for the effect of finite data sampling, of SHG signal noise and of microscopic distributions of θ_F_ and γ. Denoising filters working in the phasor space were also applied. Moreover, the results obtained by μMAPPS were validated against direct fitting of the P-SHG spectra in various samples. We adopted a widespread^20,22,27,43,60^ molecular model to assist our phasor plots analysis. Indeed, our successful application of μMAPPS to tumor tissues, where non-idealities (background, y_0_, up to 30% of the SHG signal) are present, suggests that our algorithm can be effectively extended to more complex situations taking into account axial polarization component, experimental errors and deviations from the present theoretical model^40^. With respect to existing P-SHG analysis routines^10,22,26,27,29^, μMAPPS provides a fast and intuitive representation of the structural content of the sample by working globally on the image and by grouping the pixels through non-spatial features they share with other pixels.

We applied here μMAPPS to characterize the microscopic behavior of both organized tissues, such as tendons, and of the more complex tumor ECM architecture. In this regard, three different tissue preparations (fixed sections, biopsies, *in-vivo*) have been selected for a first validation of the method. A more in depth study has been performed in tumor biopsies at two stage of progression by exploiting also geometrical features of the phasor plots and our clustering algorithm.

Our main goal was to develop and validate our phasor approach on a number of pathological relevant samples. A more refined analysis of the μMAPPS outcome in dependence of the tissue parameters, i.e. depth position, tumor species, and FOV dimensions, will be our next step in setting up a P-SHG based histopathological analysis. Here, we already pointed out the presence of a number of possible parameters for the micro-structure characterization of tissues: the geometrical features of the phasor plot (AR) and the CER factor in the cluster analysis, are two examples. For example, the biopsies at day 7 (less ordered) differ substantially from those taken at day 12 (more uniform population of clusters), notwithstanding the fact that we retrieved the same anisotropy distribution at the two time-points.

In summary, our clustering algorithm, working on a phasor-based analysis of the P-SHG images, allows to automatically back-project data in the original image highlighting areas that share the same microscopic behavior (θ_F_ and γ values within a selected threshold) and to obtain other quantitative global characterization of the microscopic architecture of tissues that can be amenable to define scores for the automatic recognition of pathologies both *ex-vivo* and *in-vivo*. We believe therefore that our method could perform fast microstructural analyses that assist or replace the histo-pathological evaluation and could open future applications for *in-situ* diagnosis of pathologies and diseases. Efforts are ongoing to develop cluster-based machine learning algorithms with diagnostic capability of tumors.

## Methods

### Two-photon microscopy set-up

The optical setup was built around a confocal scanning head (FV-300, Olympus, Japan) mounted on an upright optical microscope (BX51, Olympus, Japan), coupled to a tunable fs-pulsed Ti:Sa laser (690–980 nm, 120 fs full width at half maximum pulse duration and 80 MHz repetition frequency, Mai Tai HP, Spectra Physics, CA). A high working distance objective (NA = 0.95, WD = 2 mm, 20X, water immersion, XLUMPlan FI, Olympus, Japan) has been employed to both excite and collect the emitted signal. The backscattered second harmonic signal, primed by 800 nm wavelength, has been steered to a home-built non-descanned unit, filtered by a short-pass 670 nm filter (Chroma Inc., Brattelboro, VT, USA) and by a 400/20 nm band-pass filter (Chroma Inc., Brattelboro, VT, HQ400/20), and collected by a photomultiplier tube (HC125–02, Hamamatsu, Japan). The entire microscope is surrounded by a custom made thermostatic cabinet in which the temperature is kept at 37 °C (air thermostating by “The Cube”, Life Imaging Services, Basel, CH) for *in-vivo* measurements. For polarization-resolved SHG measurements, a half-wavelength waveplate has been placed along the optical path to control the laser polarization.

### Image acquisition and analysis

Sequences of images of single z plane have been acquired by rotating the laser polarization from 0° to 180° in steps of 5° in order to obtain a rotation of the laser polarization from 0° to 360° in steps of 10°. Each image has a resolution of 512 × 512 pixels. The acquired field of view is described in the images captions (variable between 200 × 200 μm^2^ and 600 × 600 μm^2^). The following excitation laser power, measured before the scanning-head, has been exploited: P_exc_ = 10 mW for normal mouse-tail tendon, P_exc_ = 20 mW for mouse-tail tendon in case of collagenase-digestion (due to the reduced emitted SHG signal). Regarding tumors, P_exc_ = 50 mW for histology sample analysis, while it was variable in the range 50–60 mW for biopsies and *in-vivo* experiments. Higher power (above 60 mW) have been used only during z-scan imaging deep within the tumor. P-SHG data have been acquired in the range 10–15 μm below the tumor surface. Care must be taken in the proper laser power selection in order to avoid tissue damages. Each image is the result of 3–5 Kalman average scans (512 × 512 pixels) and has been acquired in 3.4–5.6 s. For *in-vivo* P-SHG experiments in tumors, a sequence of 6 z-planes has been acquired along the z axis at 1 μm inter-distance for each incident laser polarization in order to be able to post-process the images in case of z movements. In this case 2 Kalman average scans have been acquired for each angular step. Specific corrections to the μMAPPS algorithm for birefringence, polarization cross-talk or diattenuation^36^ were not applied to our P-SHG data, collected from tissue surface (10–15 μm below the surface).

For tumor analysis, in order to further reduce the noise and retrieve the θ_F_ and γ values with an increased precision, a gaussian filter of the image stack acquired as function of θ_L_ has been implemented before μMAPPS analysis. This procedure, equivalent to a smoothing of the SHG intensity profile in dependence of θ_L_, allows us to analyze the acquired image stack pixel by pixel to retrieve θ_F_ and γ without the need of signal averaging among adjacent pixels, which can lead to a worse θ_F_ and γ maps resolution and to pixels-averaged behavior that could hide microstructural variations among them.

Moreover, following μMAPPS analysis, color-coded images are created to represent in each pixel the θ_F_ and γ values retrieved from the θ_F_ and γ p-plots, respectively.

Regarding the clustering procedure (see Supplementary Note 5), each cluster is encoded with different monochromatic LUT with an intensity scaling as the pixel integrated P-SHG signal.

The cutoff values for γ and θ_F_ used for performing the clustering analysis of the experimental data are reported in the caption of the corresponding figures. Moreover only clusters with a number of elements higher with respect a threshold (ET), computed as a percentage of the total number of analyzed pixels, have been considered and reported in the figures captions. Only P-SHG spectra above a threshold (T_NL_) are analyzed in order to discard the background contribution.

In order to extract, for each cluster, the mean SHG signal as a function of θ_L_ for the fit procedure, the signal related to each pixel of the P-SHG stack (belonging to the considered cluster) is divided for its maximum intensity and then the average of these signals is computed.

In the θp-plot, the ratio between the major and minor axis of the ellipse enclosing the points, AR, has been extracted by means of the *Measure* tool of ImageJ (U.S. National Institute of Health, Bethesda, Maryland, USA).

### Software

All the polarization-dependent analysis based on the phasor approach, the θ and γ plots, maps and histograms have been obtained by means of a custom designed C++ based software. The program Origin (Origin 8.5, OriginLab Corporation) has been employed for the polarization-dependent SHG curves fitting. All the acquired images have been visualized and linearly contrast-adjusted using ImageJ (U.S. National Institute of Health, Bethesda, Maryland, USA). Images affected by xyz drift or to other movements during *in-vivo* image acquisition have been manually post-processed and realigned by means of ImageJ and Photoshop software.

### Mice

All mice inoculated with the murine tumor cell line B16, housed under specific pathogen‐free conditions, were female on a C57bl/6 background for at least 12 generations and were used at 7–9 weeks of age. All mice, inoculated with the tumor cell line CT26, were female on a BALB/c background for at least 12 generations and were used at 7–12 weeks of age. The mice were kept in a pathogen‐free conventional animal house facility. The animal house is run by professional employees fully equipped with state‐of‐the‐art instrumentation in order to maintain the standard of animal welfare at the maximum levels. All mice were housed in individual, ventilated cages with 12 h light/dark cycles with food and water ad libitum. Experiments were performed using protocols approved by the Institutional Animal Care and Use Committee of the University of Milano‐Bicocca and by the Italian Ministry of Health or were performed under Boston Children’s Hospital IACUC approved protocols.

### Mouse-tail tendon

Mouse-tail tendons have been harvested from sacrificed C57bl/6 background mice at 8 weeks of age. The tendons have been cut into strips 3–4 cm in length and placed in a Phosphate Buffered Saline (PBS) solution at pH = 7.4 before visualization under the two-photon microscope. Then, the samples have been mounted in a chamber made of two cover-slips sealed with silicone and filled with PBS.

For collagenase-digestion, highly purified collagenase has been employed (Collagenase VIII Sigma-Aldrich C2139) in the buffer Hepes-NaOH 10 mM pH 7.4/NaCl 150 mM/KCl 5 mM/MgCl_2_ 1 mM/CaCl_2_ 5 mM, at a concentration of 5 mg/ml. Mouse-tail tendons have been incubated in the collagenase solution for 1 h at 37 °C; for SHG visualization the samples have been placed in a chamber filled with PBS solution. SHG polarization-dependent images have been acquired at the surface of the mouse-tail tendons in order to eliminate or reduce effects due to refractive-index-induced spherical aberrations.

10 FOVs (225 × 225 μm^2^) related to 4 tendons of different mice have been analyzed.

### Cells

The murine tumor cell line B16 and the CT26 cell line were cultured in IMDM‐10 complete medium: IMDM, 10% heat‐inactivated FBS (EuroClone), 2 mM l‐glutamine, 100 U/ml penicillin, 100 μg/ml streptomycin. Cells at the confluence of 70% were collected for the mice injection.

CT26 cell line (ATCC, CRL2638), a BALB/c mouse colon carcinoma, was obtained from Mario Colombo (Istituto Nazionale del Tumori, Milan, Italy).

### Tumor injection and analysis

For the *in vivo* and *ex-vivo* B16 tumor analysis, C57bl/6 mice were inoculated in the deep derma in the left flank with the tumorigenic dose of B16 tumor cells (2 × 10^6^) at Day 0. For the *in vivo* experiment, at day 7 mice were anesthetized I.P. with a Ketamine/xilazine cocktail (0.1 mL/20 g mouse), the derma was cut off and the tumor exposed and analyzed with two-photon microscopy. For the *ex-vivo* experiments at day 7 and day 12, mice were euthanized, the tumor collected and placed in PBS solution before polarized-SHG analysis. For the *ex-vivo* (biopsies) experiments, tumors have been placed in a chamber made of two cover-slips sealed with silicone and filled with PBS solution.

For the CT26 tumor analysis, BALB/c mice were inoculated in the deep derma in the left flank with the minimal tumorigenic dose of CT26 tumor cells (5 × 10^4^) at Day 0. Explanted tumors at Day 7 were embedded in OCT freezing media (Biooptica). Sections (5 μm) were cut on a Cryostat, adhered to Superfrost Plus slide (Thermo Scientific), and then imaged under the two-photon excitation microscope.

Mice subjected to tumor injection were monitored on a daily basis for signs of discomfort, including hunched posture, ruffled fur and lack of movement within the cage. The body condition score index (a qualitative assessment of an animal’s overall appearance based on its weight, muscle mass and bone prominence) was used to evaluate the welfare of the mice. Generally, mice did not present signs of distress given the short period of time between tumor injection and killing.

Regarding experiments in histology fixed sections, during our analysis we acquired five field of views (FOVs, 283 × 283 μm^2^) for each analyzed sample (3 tumors, 3 sections per tumor).

Regarding biopsies we acquired 10 FOVs (225 × 225 μm^2^) for each analyzed tumors (n = 3) both at day 7 and at day 12 after tumor inoculation.

Results from *in-vivo* microscopy are related to 2 FOVs in 2 different animals.

### Statistical method

Unless otherwise stated, results are expressed as mean ± SEM. All statistical analyses were performed in Prism 5 (GraphPad Software). Means between two groups were compared with two-tailed Student’s t-test. The value of P ≤ 0.05 was considered statistically significant. The degree of significance was assigned as follows: *for P ≤ 0.05, **for P ≤ 0.01, ***for P ≤ 0.001, and ****for P ≤ 0.0001.

### Data availability

The data that support the findings of this study are available from the corresponding authors on reasonable request.

### Code availability

The custom-written C++ software will be made available online at http://fisica.mib.infn.it/media/homepages/biofisica/bicocca-hepth/index.html.

## Electronic supplementary material

Supplementary Information
