# Author Correction: μMAPPS: a novel phasor approach to second harmonic analysis for in vitro-in vivo investigation of collagen microstructure

**DOI:** 10.1038/s41598-018-24800-6

**Published:** 2018-04-17

**Authors:** F. Radaelli, L. D’Alfonso, M. Collini, F. Mingozzi, L. Marongiu, F. Granucci, I. Zanoni, G. Chirico, L. Sironi

**Affiliations:** 1 Dipartimento di Fisica, Università degli Studi di Milano-Bicocca, Piazza della scienza 3, 20126 Milano, Italy; 2 Dipartimento di Biotecnologie e Bioscienze, Università degli Studi di Milano-Bicocca, Piazza della Scienza 2, 20126 Milano, Italy; 3 CNR - ISASI, Institute of Applied Sciences & Intelligent Systems, Via Campi Flegrei 34, Pozzuoli, NA Italy; 4 Harvard Medical School and Division of Gastroenterology, Boston Children’s Hospital, Boston, MA USA

Correction to: *Scientific Reports* 10.1038/s41598-017-17726-y, published online 12 December 2017

In the original version of this Article, Affiliations 1 and 2 were incomplete. The correct Affiliations are listed below:

Affiliation 1:

Dipartimento di Fisica, Università degli Studi di Milano-Bicocca, Piazza della Scienza 3, 20126, Milano, Italy.

Affiliation 2:

Dipartimento di Biotecnologie e Bioscienze, Università degli Studi di Milano-Bicocca, Piazza della Scienza 2, 20126, Milano, Italy.

These errors have now been corrected in the PDF and HTML versions of the Article, and in the accompanying Supplementary Material.

