# Supplementary material for: μMAPPS: a novel phasor approach to second harmonic analysis for in vitro-in vivo investigation of collagen microstructure
Source: Sci Rep. 2017 Dec 12;7:17468. doi: 10.1038/s41598-017-17726-y (PMC5727101; doi:10.1038/s41598-017-17726-y)
Supplement: Supplementary file 1 — Supplementary Information [file 41598_2017_17726_MOESM1_ESM.pdf]

Supplementary Information.

µMAPPS: a novel phasor approach to second harmonic analysis for in vitro-in vivo investigation of collagen microstructure

F. Radaelli[1], L. D'Alfonso[1], M. Collini[1,3*], F. Mingozzi[2], L. Marongiu[2], F. Granucci[2], I. Zanoni[2,4], G. Chirico[1,3], L. Sironi[1*]

1- Dipartimento di Fisica, Università degli Studi di Milano-Bicocca, Piazza della Scienza 3, 20126, Milano, Italy
2- Dipartimento di Biotecnologie e Bioscienze, Università degli Studi di Milano-Bicocca, Piazza della Scienza 2, 20126, Milano, Italy
3- CNR - ISASI, Institute of Applied Sciences & Intelligent Systems, Via Campi Flegrei 34, Pozzuoli, NA, Italy
4- Harvard Medical School and Division of Gastroenterology, Boston Children's Hospital, Boston, MA, USA

*: laura.sironi@unimib.it; maddalena.collini@mib.infn.it

Table of contents.

- Supplementary Note 1:
  - Second Harmonic Generation Theory
- Supplementary Note 2:
  - Phasor analysis
- Supplementary Note 3:
  - Fitting method
  - y_0 effect
- Effect of data sampling on the phasor analysis.
- Effect of noise on the phasor analysis.
- Effect of the micro-structure in-homogeneity.
- Supplementary Note 4:
  - Denoising effect
- Supplementary Note 5:
  - Coherence Density Peak Clustering
- Supplementary Note 6:
  - Mouse-tail tendon

Supplementary Note 1

Second Harmonic Generation Theory

Second Harmonic generation (SHG) is a nonlinear coherent optical process where two incident photons of frequency ω are converted into a single photon of exactly twice the frequency 2ω. SHG offers several advantages with respect to other imaging techniques: it avoids the need of exogenous labels and it is generated by means of infrared laser, thereby increasing the penetration depth into tissue and reducing sample damage. Since it is a second-order nonlinear process, SHG provides intrinsic optical sectioning related to the dependence of SHG intensity to the square of the excitation intensity. SHG does not suffer of photobleaching and phototoxicity since it a nonlinear scattering process, not relying with absorption phenomena. Moreover, SHG is restricted to molecules with non-centrosymmetric organization, resulting in a high sensitivity to polarization-dependent measurement. This property can be exploited to provide quantitative information on the local molecular structure (and/or organization) of the sample and to directly probe the assembly of collagen into tissues. Indeed, numerous studies have demonstrated that collagen, myosin and microtubules in cells and tissues can be non-invasive imaged by exploiting SHG microscopy.

Polarization-dependent method detects the SHG signal as a function of the relative angle between the laser polarization and the unknown direction of the SHG emitters, allowing to probe their molecular distribution and therefore to reconstruct the structure of the investigated sample. In order to extract the molecular organization, we must consider proper theoretical model relating the collagen structure and the SHG optical principles.

The total polarization for a medium interacting with an incident electric field **E** at frequency ω is[61,62]:

$$P_i = P_i^{(0)} + \sum_j \chi_{ij}^{(1)} E_j + \sum_{jk} \chi_{ijk}^{(2)} E_j E_k + ... \qquad \text{(S1)}$$

where i, j and k denote vector or tensor component indices, $\mathbf{P}^{(0)}$ is the static intrinsic polarization, and $\chi_{ij}^{(1)}$ are the components of the linear electric susceptibility of the medium. SHG, a degenerate case of three-wave mixing where $\omega_1 = \omega_2 = \omega$, is usually described by using second-order nonlinear optical susceptibility tensor $\chi^{(2)}$, which represents the macroscopic (0.1-1 μm) nonlinear response of the medium composed of elementary scatter elements at a microscopic molecular level. In general, the second-order nonlinear optical susceptibility tensor $\chi^{(2)}$ is a third-rank tensor with (3 × 3 × 3) elements and the maximum number of independent components of $\chi^{(2)}$ is 18 due to the following symmetry:

$$\chi_{ijk}^{(2)}(\omega,\omega) = \chi_{ikj}^{(2)}(\omega,\omega) \qquad \text{(S2)}$$

The second order polarization can be expressed as:

$$P_i^{(2)}(2\omega) = \sum_{j,k} \chi_{ijk}^{(2)}(\omega,\omega) E_j(\omega) E_k(\omega) \qquad \text{(S3)}$$

Several theoretical models of the local second–order nonlinear susceptibility tensor $\chi^{(2)}$ have been proposed[12,21,27,33,40,43,62-64] for interpreting the polarization-dependent second harmonic generation (P-SHG) signal. In all the samples capable to produce SHG signal (muscle, microtubules, collagen in animals and starch and cellulose in plants), the SHG active macromolecular assemblies are usually described by hexagonal (C_6) or cylindrical (C_∞) symmetry, which are equivalent within the

Kleinman conditions (that implies that no net energy absorption is involved)[65]. Under these assumptions it is possible to further reduce the number of the $\chi^{(2)}$ components from 18 to 2.

By considering the coordinate system in which the sample lies in the xz-plane and the propagation direction of the laser beam is along the y-direction (see Supplementary Fig. 1), if the hexagonal symmetry is applied, the non-zero $\chi^{(2)}$ tensor elements follows the conditions:

$$\chi^{(2)}_{zzz},\ \chi^{(2)}_{zxx} = \chi^{(2)}_{zyy},\ \chi^{(2)}_{xxz} = \chi^{(2)}_{xzx} = \chi^{(2)}_{yzy} = \chi^{(2)}_{yyz} \tag{S4}$$

$$\chi^{(2)}_{xzy} = \chi^{(2)}_{xyz} = -\chi^{(2)}_{yxz} = -\chi^{(2)}_{yzx}$$

If Kleinman symmetry is assumed, the entries of the tensor identified by a permutation of indices (i,j,k) have the same value[65].This results in seven nonzero components and two independent tensor components:

$$\chi^{(2)}_{xxz} = \chi^{(2)}_{xzx} = \chi^{(2)}_{yzy} = \chi^{(2)}_{yyz} = \chi^{(2)}_{zyy} = \chi^{(2)}_{zxx} \text{ and } \chi^{(2)}_{zzz} \tag{S5}$$

If now an electric field propagating along the z-axis and linearly polarized at an angle (θ_L-θ_F) with respect to the fibril axis (axis of cylindrical symmetry) is considered:

$$\vec{E} = E\sin(\theta_L - \theta_F)\hat{e}_x + E\cos(\theta_L - \theta_F)\hat{e}_z \tag{S6}$$

the second-order polarization can be written as:

$$\vec{P}^{(2)} = \left[2\chi^{(2)}_{zxx}E\sin(\theta_L - \theta_F)E\cos(\theta_L - \theta_F)\right]\hat{e}_x + [\chi^{(2)}_{zxx}(E\sin(\theta_L - \theta_F))^2 + \chi^{(2)}_{zzz}(E\cos(\theta_L - \theta_F))^2]\hat{e}_z \tag{S7}$$

Therefore, the total SHG intensity, proportional to $\left|\vec{P}^{(2)}\right|^2$, is given by:

$$I_{SHG}(\vartheta_L) = k\left\{\sin^2[2(\theta_L - \theta_F)] + [\sin^2(\theta_L - \theta_F) + \gamma\cos^2(\theta_L - \theta_F)]^2\right\} \tag{S8}$$

where θ_L and θ_F are, respectively, the laser excitation polarization angle and the fibril orientation angle with respect to a fixed direction in the laboratory frame (axis x in Supplementary Fig. 1). These relations are valid since the collagen fibrils lie parallel to the surface of the tissue (the collagen fibrils and the incident electric field are both within the focal plane). The scale factor k includes the absolute intensity of the SHG signal and the setup parameters (e.g. laser power, optical transmission ratios for both excitation and signal light paths, detector sensitivity).

From equation (S8) it is therefore possible to retrieve: (i) the angle θ_F of the collagen fibrils axis with respect to the laser excitation polarization direction at θ_L; (ii) the ratio $\gamma = \frac{\chi^{(2)}_{zzz}}{\chi^{(2)}_{zxx}}$, which reflects the anisotropy of the nonlinear response of collagen fibrils. The parameter γ is ≈0.5 for myosin and ≈1.5-2 for collagen[27,40,26,54].

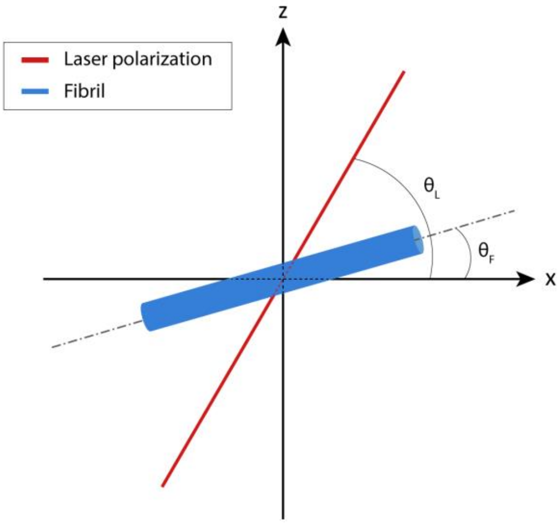

Supplementary Figure 1. Coordinate system scheme: the laser is incident along the y-direction, while the collagen fibrils lie in the xz-plane. θ_L and θ_F are, respectively, the laser excitation polarization angle and the fibril orientation angle with respect the x-axis direction.

Supplementary Note 2

Phasor analysis.

Supplementary Figs. 2 and 3 (left side panels) show examples of simulated I_{SHG} profiles for samples characterized by different values of the parameter γ and of the angle θ_F.
By applying the phasor approach, the normalized polarization-dependent SHG (P-SHG) curve is transformed into a point in the phasor plot, as shown in the right side panels of Supplementary Figs. 2 and 3. The coordinates of the point are represented by the real and the imaginary parts of the first harmonic Discrete Fourier Transform of the normalized P-SHG curve.
In particular, two phasor plots, the θ and γ p-plots, were created to extract the θ_F and the γ values without fitting procedures.

In the θp-plot, the coordinates (g_θ, s_θ) of the point are the cos/sin first Discrete Fourier Transform (DFT) of the corresponding normalized P-SHG curve, $\{I(\theta_L^n)\}_{n=0..N-1}$, acquired as a function of θ_L($0 \leq \theta_L < 3\pi/2$) with resolution $\Delta\theta$ ($N=\pi/\Delta\theta$and $\Delta\theta=10^o$):

$$g_\theta = \frac{\sum_{n=0}^{N-1} I(\theta_L^n)\cos(\theta_L^n K_\theta)}{\sum_{n=0}^{N-1} I(\theta_L^n)} \quad ; \quad s_\theta = \frac{\sum_{n=0}^{N-1} I(\theta_L^n)\sin(\theta_L^n K_\theta)}{\sum_{n=0}^{N-1} I(\theta_L^n)} \qquad \text{(S9)}$$

where $K_\theta = 2\pi(N\Delta\theta)^{-1}$. The coordinates ($g_\gamma$,$s_\gamma$) in the second γp-plot are defined as

$$g_\gamma = \frac{\sum_{n=0}^{N/2-1} I(\theta_L^n+\theta_F)\cos((\theta_L^n+\theta_F)K_\gamma)}{\sum_{n=0}^{N-1} I(\theta_L^n+\theta_F)} \quad ; \quad s_\gamma = \frac{\sum_{n=0}^{N/2-1} I(\theta_L^n+\theta_F)\sin((\theta_L^n+\theta_F)K_\gamma)}{\sum_{n=0}^{N-1} I(\theta_L^n+\theta_F)} \qquad \text{(S10)}$$

where $K_\gamma = 2\pi[N(\Delta\theta+\theta_F)]^{-1}$. In this case the first DFT is computed in the $\theta_F \leq \theta_L \leq \theta_F + \pi/2$range with θ_F retrieved from the θp-plot.

Supplementary Fig. 2 shows four different I_{SHG} profiles as a function of the laser polarization angle θ_L, characterized by different values of the anisotropy parameter γ (γ=0.5, γ=1.0, γ=1.5, γ=3.0) and θ_F=0. Differences in γ values for P-SHG signals characterized by the same angle θ_F affect the radial position of the corresponding points in the θp-plot. The point characterized by γ=1 lies in the center of the θp-plot, while the point with γ=0.5 is in a π-tilted position with respect to that with γ>1 (see also Fig. 2b of the Main text). In the γp-plot, the position of the points depends only on the value of γ and it is not affected by θ_F.

Three different I_{SHG} profiles as a function of θ_L, obtained for a fixed γ=1.5 and different θ_F parameter (θ_F=30°, θ_F=60°, θ_F=120°), are shown in Supplementary Fig. 3. In the θp-plot therefore they all lie on the same circle but at different angular position, while in the γp-plot the points are superimposed due to their shared γ value.
It is clear that the phasor approach applied to the acquired polarized-dependent SHG curves can be exploited to access information on the structural organization and distribution of SHG emitters within the sample.

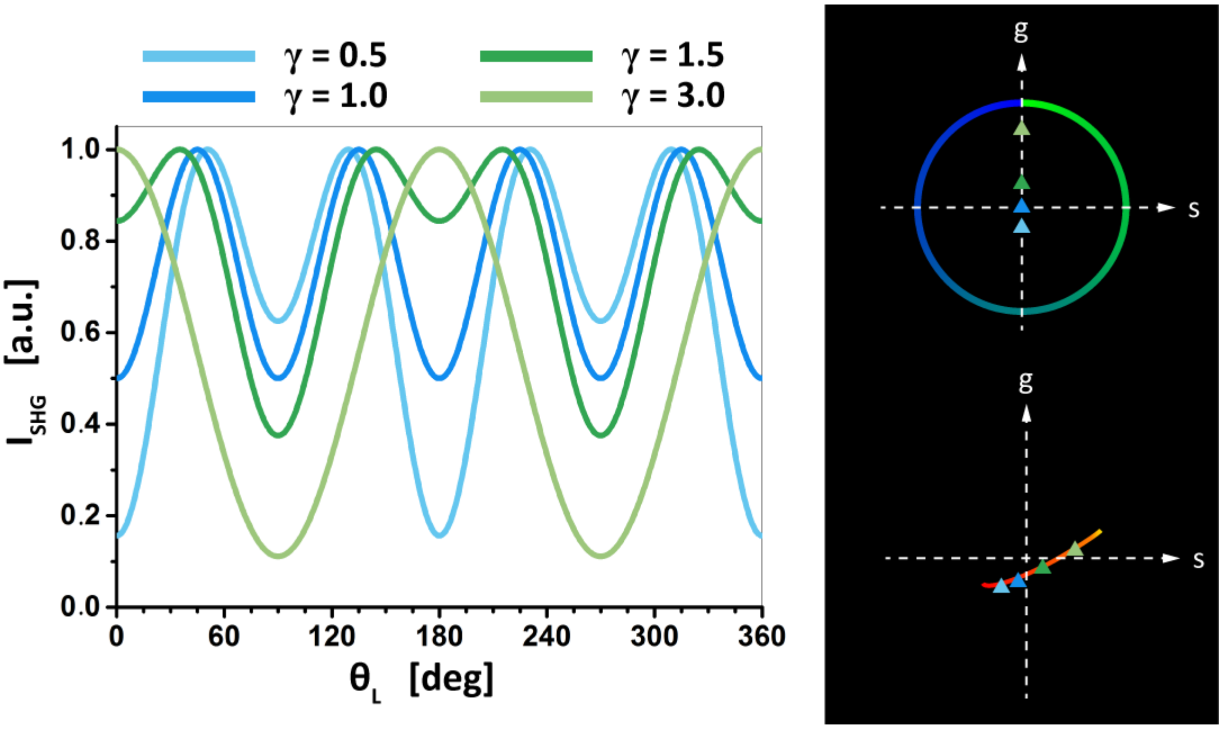

Supplementary Figure 2. Left panel: four different I_{SHG} normalized curves, rescaled to their respective maximum, are drawn as a function of the laser polarization angle θ_L. The simulation parameters are: γ=0.5, γ=1.0, γ=1.5, γ=3.0, while θ_F=0, fixed for all the curves. Right upper panel: triangles in the θp-plot represent the position of the points obtained by Fourier transforming (equation (S9)) the simulated P-SHG curves (equation (S8)). The triangles lie along the same direction since they are characterized by the same value θ_F=0, while their radial positions are due to the different γ values. The green-blue color coded circle is the reference curve obtained by simulating equation (S8) (representing the theoretical model) within the [0,π] angular range and $\gamma \rightarrow \infty$. Right lower panel: triangles in the γp-plot represent the position of the points obtained from the simulated P-SHG curves that are Fourier transformed by means of equation (S10) with θ_F retrieved from the θp-plot. The different positions of the triangles are due to the different γ values. The red-yellow color coded reference curve is obtained by simulating equation (S8) within the $[\theta_F,(\theta_F+\pi/2)]$ angular range while varying γ from 0 to 10.

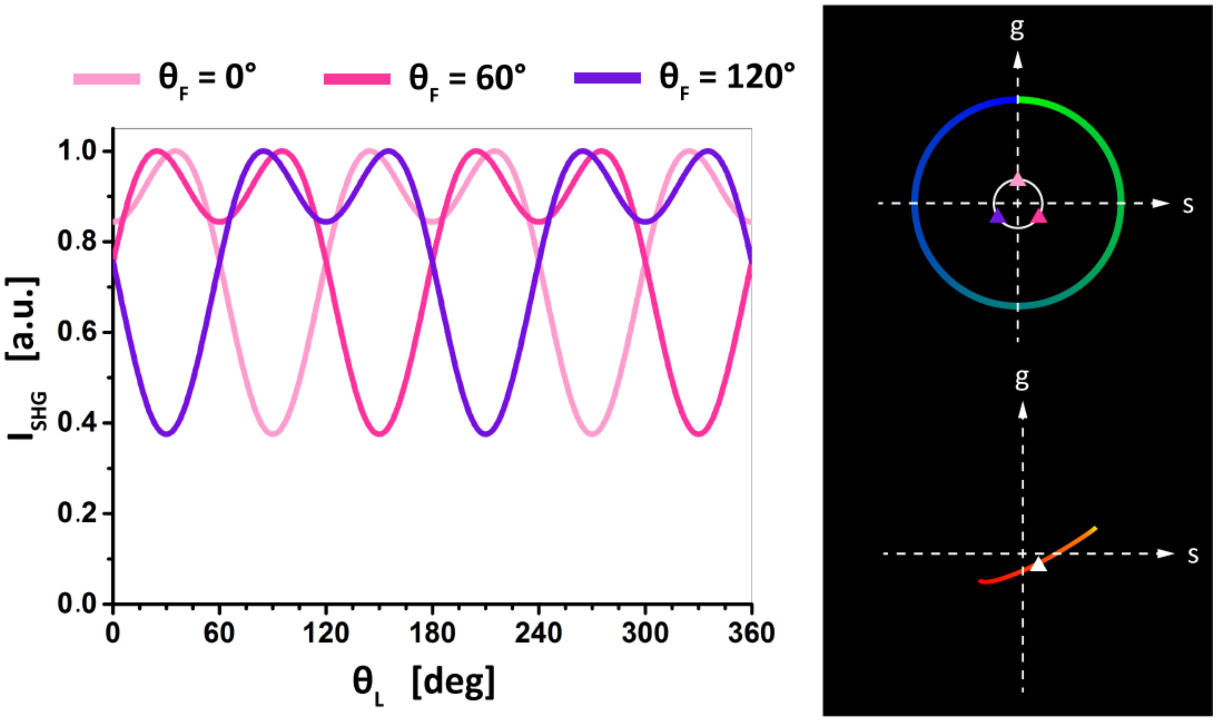

Supplementary Figure 3. Left panel: three different I_{SHG} normalized curves, rescaled to their respective maximum, are drawn as a function of the laser polarization angle θ_L. The simulation parameters are: θ_F=30°, θ_F=60°, θ_F=120°, while γ=1.5, fixed for each curve. Right upper panel: triangles in the θp-plot represent the position of the points, obtained by Fourier transforming the simulated P-SHG curves (equation (S8)) by means of equation (S9). They all lie on the same circle since they are characterized by the same γ values, while their position on the circle is associated to the different θ_F values. The green-blue color coded circle is the reference curve obtained by simulating equation (S8) (representing the theoretical model) within the [0,π] angular range and $\gamma \to \infty$. Right lower panel: triangles in the γp-plot represent the positions of the points obtained by Fourier transforming the simulated P-SHG curves by means of equation (S10) with θ_F retrieved from the θp-plot. The triangles are superimposed since they are characterized by the same γ=1.5 value. The red-yellow color coded reference curve is obtained by simulating equation (S8) within the $[\theta_F,(\theta_F+\pi/2)]$ angular range while varying γ from 0 to 10.

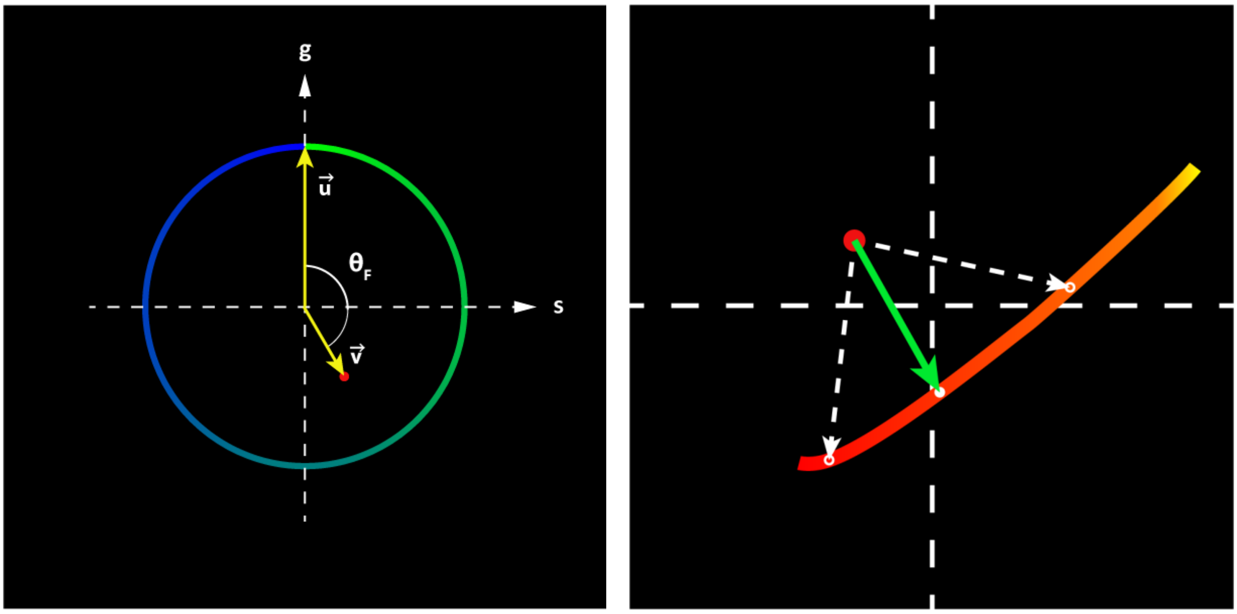

Supplementary Figure 4. Left panel: extraction of θ_F from the θp-plot. θ_F is the angle subtended between the yellow vectors $\vec{v} = (s_i, g_i)$ and $\vec{u} = (0,1)$ lying along the direction of $\theta_F = 0$. s_i and g_i represent the coordinates of the point under investigation (represented in red) in the phasor plot. Right panel: the parameter γ associated to the experimental red point is obtained by retrieving the minimum Euclidean distance projection onto the Reference Curve, as defined by equation (5) (Main text). The distances between the considered experimental point and points of the γ reference curve are computed, as shown by the white dashed arrows, searching for the minimum represented here by the green arrow. It must be noted that the γ reference curve is obtained with the θ_F value retrieved from the first phasor plot and with the experimental sampling angle Δθ. We refer to Supplementary Fig. 5b for a description of the algorithm used to derive the γ parameter when the microscopic model must take into account a non negligible background level (see Equation (S11)).

Supplementary Note 3

Fitting method

In order to compare the data obtained by means of the phasor approach with those retrieved with a fitting procedure, we use the following function:

$$I_{SHG} \propto k\left\{\sin^2[2(\theta_L - \theta_F)] + [\sin^2[(\theta_L - \theta_F) + \gamma \cos^2(\theta_L - \theta_F)]^2\right\} + y_0 \quad \text{(S11)}$$

Where θ_L and θ_F are the laser excitation polarization angle and the fibril orientation angle, respectively, with respect to a fixed direction in the laboratory frame (axis x in Supplementary Fig. 1). This relation is valid since the collagen fibrils lie parallel to the surface of the tissue (the collagen fibrils and the incident electric field are both within the focal plane). The scale factor k includes the absolute intensity of the SHG signal and setup parameters (e.g. laser power, and optical transmission ratios for both excitation and signal light paths and for the detector sensitivity).

The parameters k, γ, θ_F and y_0 were set free in the fitting algorithm, based on a nonlinear least-squares fitting routine (Origin 8.5, OriginLab Corporation) using over five hundred iterations per pixel. The parameter y_0, added in other analysis based on fit procedure[40,60], includes experimental errors and any eventual deviation from the theoretical model, so it is not to be considered a simple background contribution[40,60]. y_0 suffers also of pixel to pixel slight difference.

The fitting function was used to interpolate the SHG signal as a function of the laser polarization angle θ_L, as benchmark for μMAPPS. The half-waveplate was rotated from 0° to 180° in steps of 5° in order to obtain a rotation of the laser polarization from 0° to 360° in steps of 10°. From the fitting procedure the parameters k, γ, θ_F and y_0 are retrieved.

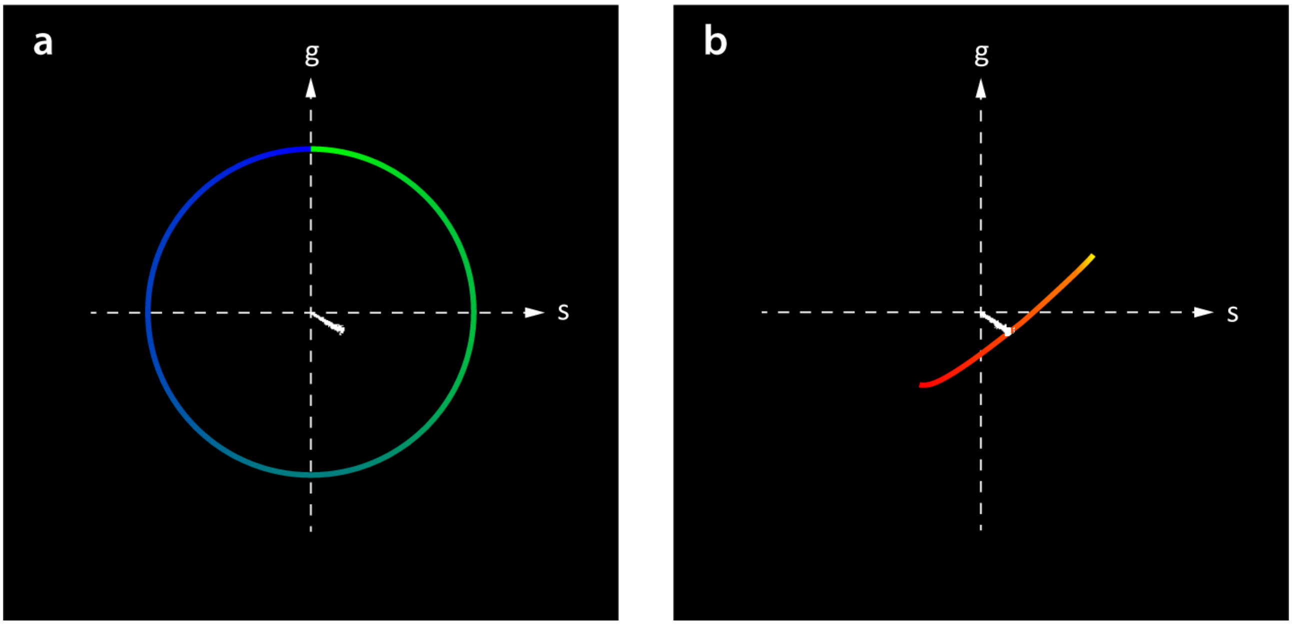

Supplementary Figure 5. "Background y_0" effect on θ and γp-plots. The term y_0, exploited in the fitting-based procedure, includes both experimental errors and deviation from the selected theoretical model. In tumor samples, collagen fibrils can no longer be considered to lie parallel to the surface of the tissue. Therefore, an addition term y_0, accounting for an angular distribution of collagen fibrils within each pixels, has to be examined. Simulations have been performed in order to evaluate the position of the points derived from the DFT of the P-SHG curves in the θ and γp-plots.

Data were simulated by substituting in equation (S11) (which is equal to equation (S8) with the additional term y_0) the parameters k=1000, θ_F=60°, γ=1.5 and y_0 variable in the range [0, 100000]. A Gaussian noise distribution has been superimposed to the SHG signal (I_{SHG}), with a standard deviation of $5\sqrt{I_{SHG}}$ and mean I_{SHG}. The θ reference curve in a) has been obtained by simulating equation (S8) within the [0,π] angular range and γ –> ∞, while the γ reference curve in b) has been obtained by simulating equation (S8) within the $[\theta_F, (\theta_F + \pi/2)]$ angular range while varying γ from 0 to 10. 10000 points were sampled for the simulation. When y_0 increases, the θ and γ spectra move towards the coordinate (0,0) in the θ and γp-plots along a straight line, as shown in a) and b) respectively. As shown in a), following this effect, a point initially lying on the reference circle characterized by θ_F =60° and γ=1.5 will progressively move on a circle nearest to the center of the θp-plot, while maintaining the same θ_F value. b) shows the movement of the points towards the center of the γ p-plot as y_0 increases. Since, due to the y_0 effect, the γ values can no longer be retrieved by means of equation (5) (Main text), a modified procedure has been devised. We computed the angles between the following vectors: 1) the normalized vector pointing from the center of the γp-plot to the coordinates of the considered point; 2) the vector pointing from each point of the reference curve to the coordinates of the considered point. The γ value of the considered pixel is assumed equal to the γ value of the point on the reference curve for which this angle is minimum.

Points that, following this procedure, are associated with γ values lying outside the range [0,10] are discarded from further analysis and not reported in the θ and γ maps.

Effect of data sampling on the phasor analysis.

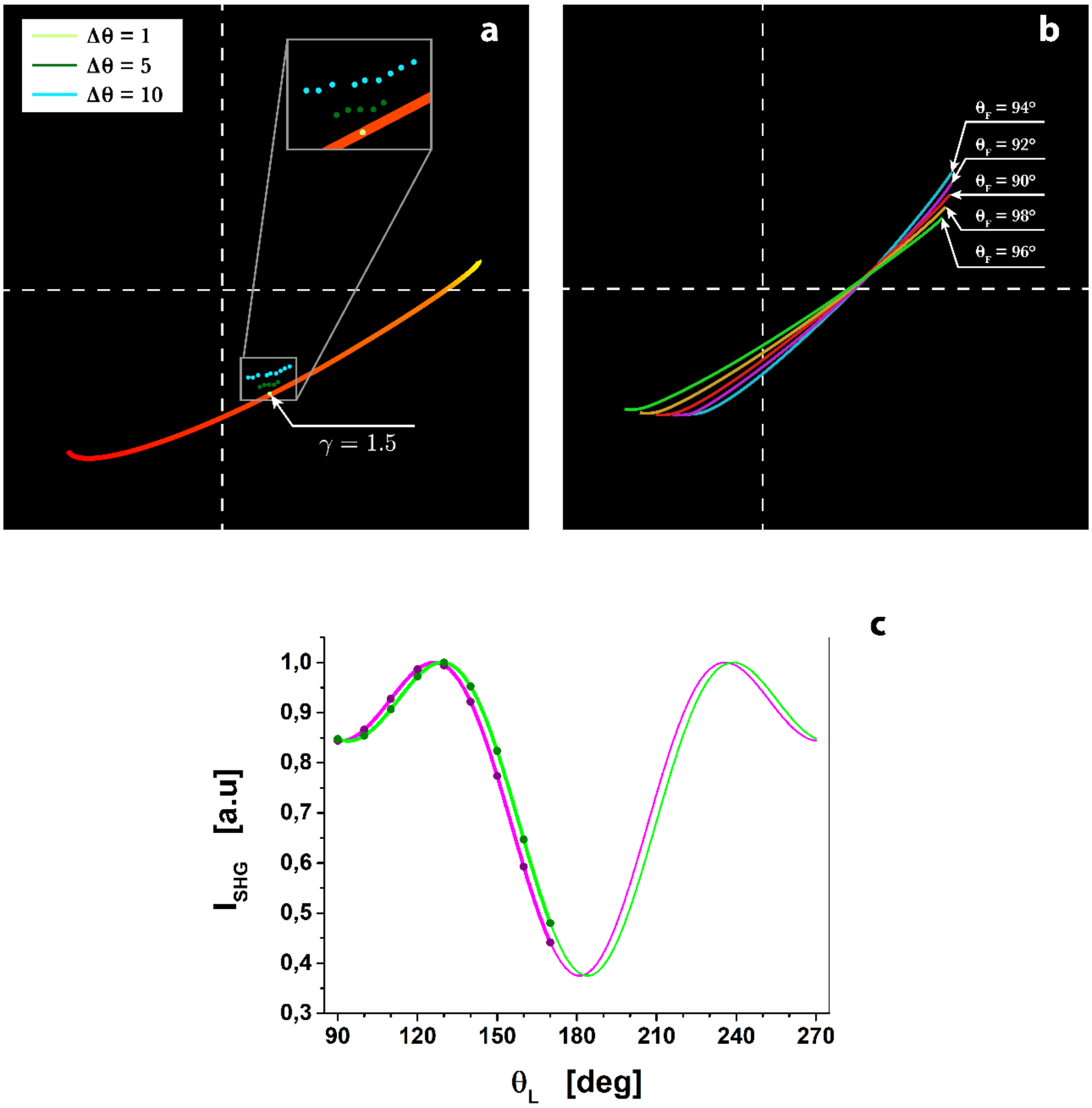

Supplementary Figure 6. Effect of data sampling on the γ-spectrum in the γp-plot. a) The reference curve (represented in red-yellow color code) and therefore the position of the points in the γp-plot are dependent on both θ_F and $\Delta\theta$ parameters. The image shows the effect of the data sampling $\Delta\theta$when different values of θ_F =90°, 91°, 92°, 93°, 94°, 95°, 96°, 97°, 98°, 99° with the same γ=1.5 are considered. When $\Delta\theta$=1°, points corresponding to different values of θ_F lie in the same position on the shown reference curve (obtained considering $\Delta\theta$=1°, yellow dot in panel a). $\Delta\theta$=5° means that the reference curve is simulated starting from P-SHG spectra sampled every 5°. The projections of data simulated for different θ_F values lie in this case on five different reference curves obtained by substituting θ_F =90°,91°,92°,93° or 94° in equation (S10) (each passing through one of the five green dots in panel a). Two points characterized by two different values of θ_F, whose absolute difference is an integer multiple (n) of $\Delta\theta$, lie on the same reference curve. Let us consider θ_{F1}- θ_{F2}=95°-90°=n$\Delta\theta$=5°: in this case the point with θ_F =95° will be superimposed to the point with θ_F =90° on the same reference curve. In a similar way, also the couples of points 96°-91°, 97°-92°, 98°-93°,99°-94° will be superimposed, each of them lying on their respective reference curve. For $\Delta\theta$=10° a similar approach can be followed: $\Delta\theta$=10° implies that the considered points can lie on ten different reference curves, which in this case are obtained by substituting θ_F =90°,91°,92°,93°,94°,95°,96°,97°,98° or 99° in equation (S10) (see blue dots in panel a). Five of these reference curves are shown in panel b). Panel c) shows an intuitive explanation of the data sampling effect for two curves characterized by θ_{F1}=91° (magenta) and θ_{F2}=94°

(green), with γ=1.5 and $\Delta\theta$=10°. Since θ_{F1}- θ_{F2}≠n$\Delta\theta$ (and γ_1=γ_2), the Discrete Fourier Transform will be calculated starting from the nearest experimentally sampled angle, which is θ_F=90°and different portion of the P-SHG curves will be therefore analyzed (shown by the increased thickness of the curves) to obtain the (g_γ,s_γ) coordinates by equation (S10). This results in different γ-reference curves in dependence on both $\Delta\theta$ and θ_{F1} values. Instead, if θ_{F1}- θ_{F2}=n$\Delta\theta$, the same portion of the P-SHG curve is DFT analyzed since in this case θ_{F1} and θ_{F2} angles are characterized by the same distance from the nearest experimentally sampled θ_F angle.

Effect of noise on the phasor analysis.

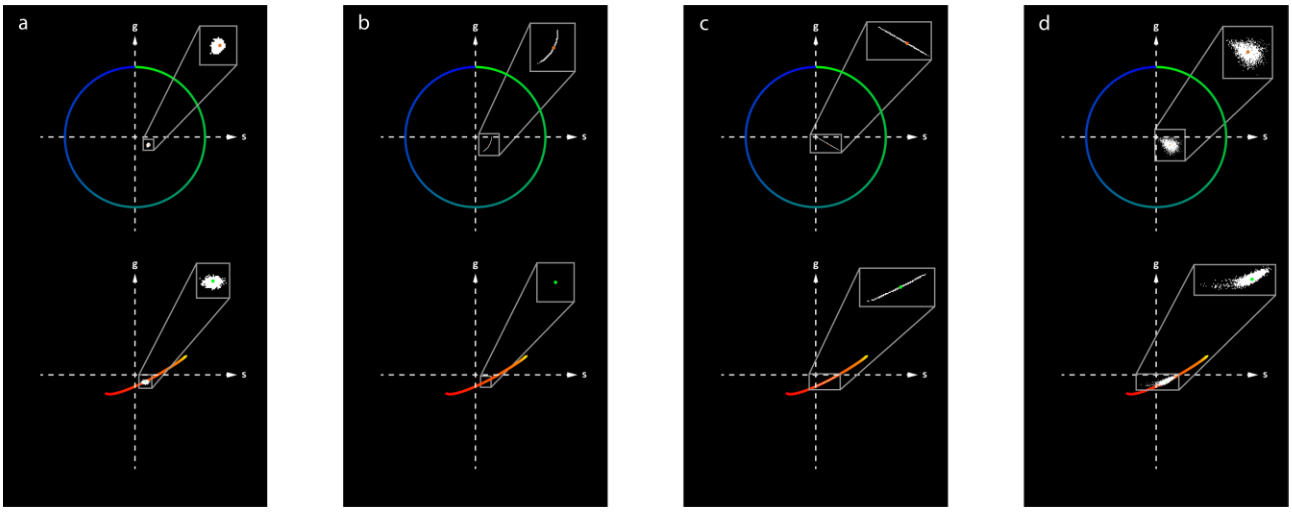

Supplementary Figure 7. Effect of noise on the distribution of points in the θ and γp-plots. a) data were simulated by substituting in equation (S8) the parameters k=1000, y_0=0, θ_F=60° and γ=1.5 with a Gaussian noise distribution, superimposed to the SHG signal (I_{SHG}), characterized by a standard deviation of $5\sqrt{I_{SHG}}$ and mean I_{SHG}, where the proportional constant 5 has been experimentally obtained and depends on the photomultiplier gain. b) data were simulated by substituting in equation (S8) the parameters k=1000, y_0=0, γ=1.5, and a Gaussian distribution of θ_F angle among the pixels of the image was assumed, with a mean angle $\bar{\theta}_F = 60°$ and a standard deviation $\sigma_{\bar{\theta}_F} = 5°$ .c) data were simulated by substituting in equation (S8) the parameters k=1000, y_0=0, θ_F=60°, and a Gaussian distribution of the γ parameter among the pixels of the image was assumed, with a mean $\bar{\gamma} = 1.5$ and a standard deviation $\sigma_{\bar{\gamma}} = 0.15$. d) The effect of the three distributions are considered together. Data were simulated by substituting in equation (S8) the parameters k=1000, y_0=0, and a Gaussian distribution among the pixels of the image was assumed for both θ_F and γ ($\bar{\theta}_F = 60°$ and $\sigma_{\bar{\theta}_F} = 5°$, $\bar{\gamma} = 1.5$ and $\sigma_{\bar{\gamma}} = 0.15$) together with a Gaussian noise distribution of the SHG signal (standard deviation $5\sqrt{I_{SHG}}$ and mean I_{SHG}).

The orange dots in the θp-plots and the blue dots in the γp-plots represent the distribution of points without the application of the respective Gaussian noise and/or θ_F and γ Gaussian distributions. 2500 points were sampled for each simulation.

Effect of the micro-structure in-homogeneity.

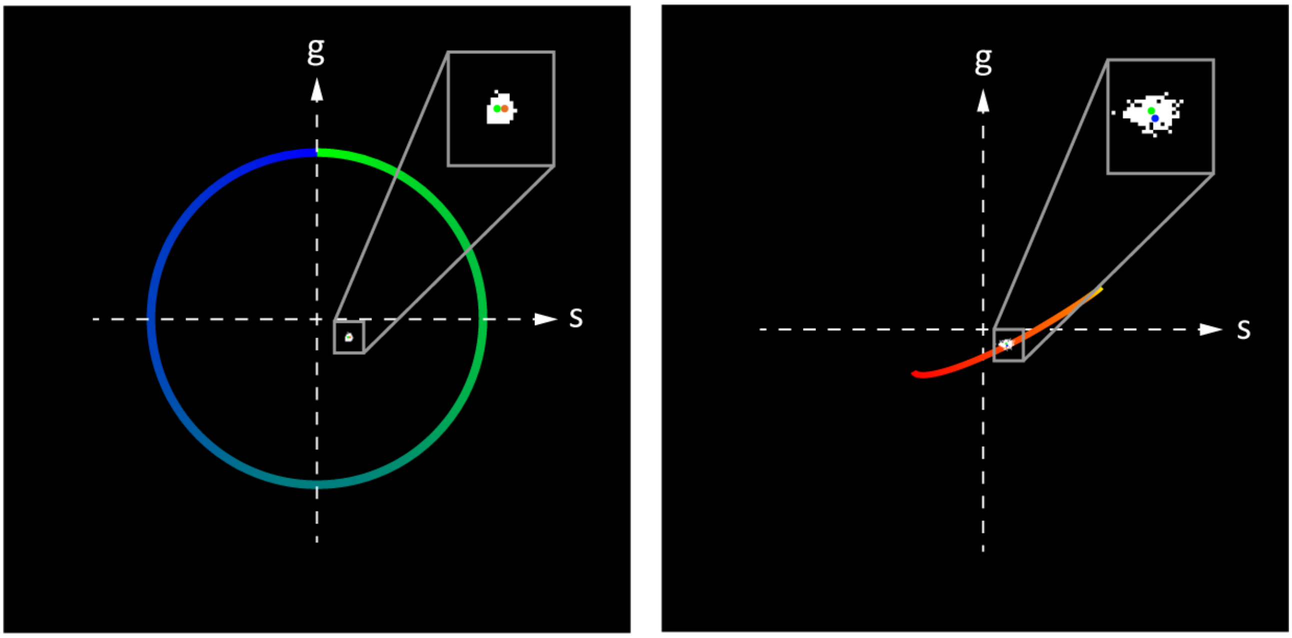

Supplementary Figure 8. Detailed effect of the fibrils angle distribution within each pixel of the image and of the simultaneous superimposed Gaussian noise on the points in the θ and γp-plots. Data with and without the application of the θ_F angular distribution and data obtained with the simultaneous superposition of a Gaussian noise to the θ_F distribution are reported. The orange (θp-plot) and blue (γp-plot) dots represent data simulated by substituting in equation (S8) the parameters k=1000, y_0=0, $\theta_F = 60°$ and γ=1.5. The green dots represent the simulated output when a Gaussian distribution of the θ_F angle (with a mean angle $\bar{\theta}_F = 60°$ and a standard deviation $\sigma_{\bar{\theta}_F} = 5°$)within each pixel was added to the simulated image. White dots represent data in which a Gaussian noise distribution on the intensity, characterized by a standard deviation of $5\sqrt{I_{SHG}}$ and mean I_{SHG}, was considered together with the previous θ_F angular distribution. 2500 points were exploited for each simulation.

Supplementary Note 4

Denoising effect

The deleterious noise effects on the P-SHG signals can be reduced by processing the matrices representing the image in the phasor space. These matrices have the same dimension of the original images acquired at different θ_L. The entries are the real or imaginary components of the first harmonic Discrete Fourier Transform (DFT) of the normalized signal from individual pixels in the stack of images. It is then clear that a couple of matrices, containing respectively real and imaginary values of the DFT, is associated to the θp-plot and another couple to the γp-plot.

Denoising is realized by applying a median filter to each matrix separately, which yields to a reduction of the scatter of points around the proper position in the phasor plot while preserving the original image resolution.

The matrices associated to the two phasor plot are not denoised at the same time. Since the first harmonic DFT of the normalized P-SHG signal between [0,π] is employed in the calculation of θ_F, fundamental for performing the second Discrete Fourier Transform, the matrices for the first phasor plot are processed before estimating θ_F, then the last two matrices are calculated, processed and used for obtaining the value of γ.

In the experiments reported here a 3x3 pixels median filter has been exploited. Moreover, the filtering procedure has been repeated five times (since it has been proved that repeated application lead to a plateau of improvement in denoising[55]).

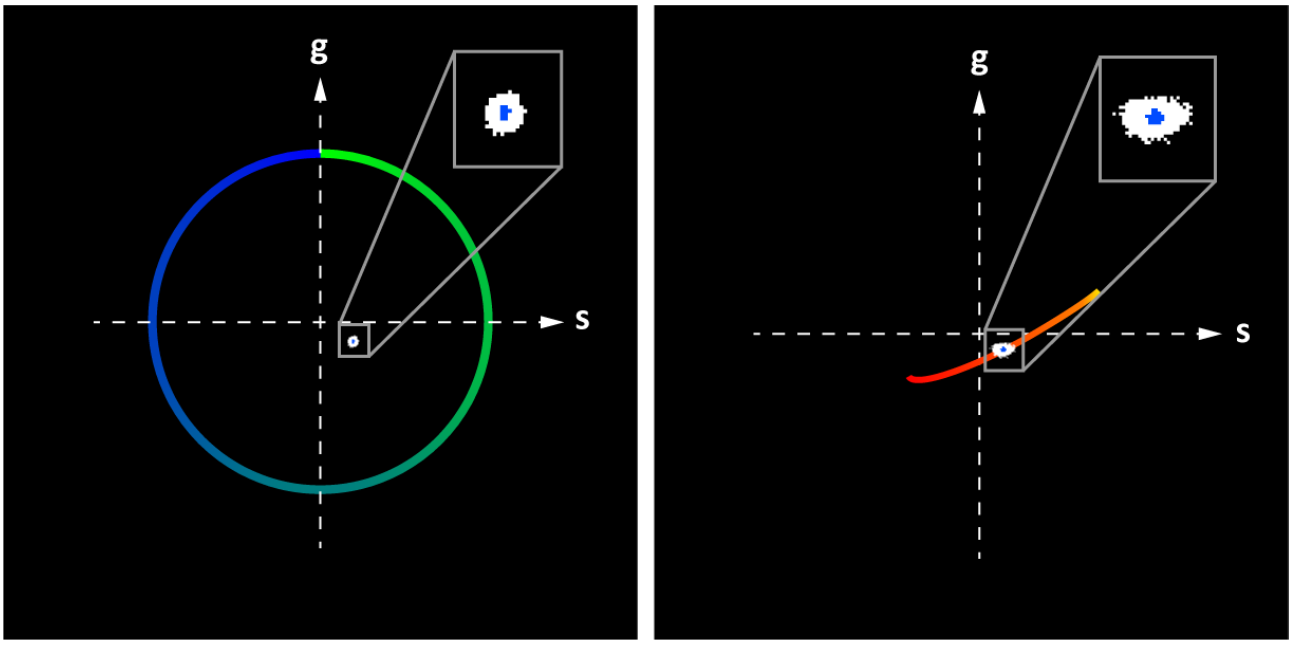

Supplementary Figure 9. Effect of denoising on simulated data distribution in the θ and γp-plots. White dots represent data obtained by considering a Gaussian noise distribution (standard deviation of $5\sqrt{I_{SHG}}$ and mean I_{SHG}) in the SHG signal, given by equation (S8). The free parameters in equation (S8) have been fixed to: k=1000, y_0=0, $\theta_F = 60°$ and γ=1.5. 2500 spectra were sampled for the simulation. The denoise is obtained by applying five times a 3x3 pixels median filter to the s and g coordinates of the points in the phasor space. As can be seen from the figure, the effect of the filter is to reduce the scatter of points in both phasor plots.

Indetermination zone

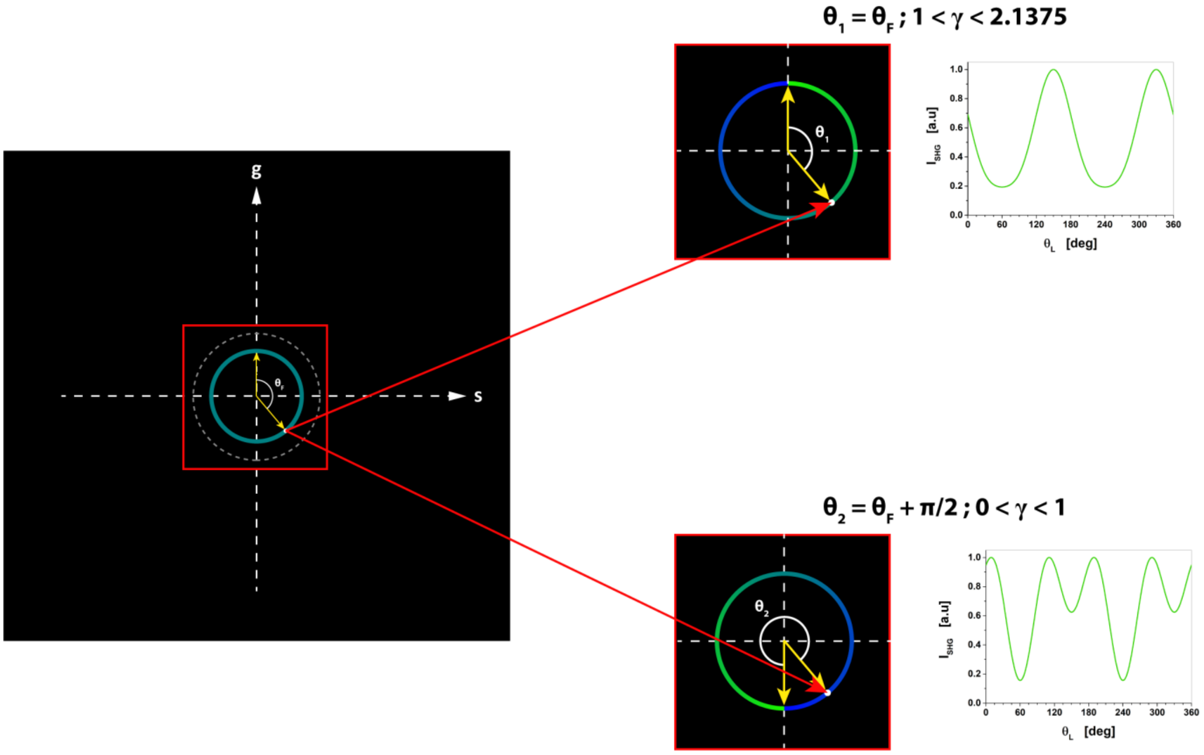

Supplementary Figure 10. Main panel, left. The area enclosed between the white dashed circle (corresponding to γ=0 or γ=2.1375) and the center of the reference system (γ=1) represents the indetermination zone. A point lying on an indetermination circle (one example shown in cyan color) in the θp-plot can be characterized by two different sets of parameters: a) $\theta_1=\theta_F$ and $1<\gamma<2.1375$ or b) $\theta_2=\theta_F+\pi/2$ and $0<\gamma<1$, with θ_1 and θ_2 computed as shown in the figure. The panels on the right report the rescaled second harmonic intensity curves as a function of the laser polarization, obtained by substituting the two sets of parameters in equation (S8), showing a clear difference in the shape of the P-SHG spectrum.

Supplementary Note 5

Coherence Density Peak Clustering

We exploited a custom clustering algorithm in order to highlight regions in the sample related to different micro-structural parameters. This algorithm, based on the maximum density approach, works in the (θ_F,γ) space where each pixel is projected. It starts by calculating, for each pixel, the density ρ_i:

$$\rho_i = \sum_j \chi(\Delta\vartheta_{ij} - \theta_c)\chi(\Delta\gamma_{ij} - \gamma_c) \qquad \text{(S12)}$$

where the sum is performed over any other pixels. χ is the Heaviside step function and θ_C and γ_C are arbitrary chosen cut-off. $\Delta\gamma_{ij}$ is the absolute difference between the values of γ for pixels i and j, while $\Delta\theta_{ij}$ is the angle between the two vectors representing the same pixels in the θp-plot. We order the pixels according to their density and apply the following iterative procedure to obtain the cluster center. i) The first unprocessed pixel with highest density is hypothesized as the cluster center and marked as processed. ii) All the unprocessed pixels within θ_C and γ_C from the putative center and with lower density are marked as processed and excluded from the list of the putative cluster centers. iii) The procedure is iterated until every pixel is marked as processed.

The pixels who are not a center are assigned to the j-th cluster for which the pixel-center distance (equation (S13)) is the lowest. Provided that $\Delta\theta_{ij}$ and $\Delta\gamma_{ij}$ are within the respective cutoff values, the following definition of distance has been employed:

$$d_{ij} = \sqrt{\left|\frac{\Delta\vartheta_{ij}}{\theta_C}\right|^2 + \left|\frac{\Delta\gamma_{ij}}{\gamma_C}\right|^2} \qquad \text{(S13)}$$

Moreover, if the number of pixels grouped in a cluster at the end of the procedure is lower than a user selected threshold, the cluster is discarded and its pixels are again marked as unprocessed. At this stage, each unprocessed pixel is re-assigned to the nearest cluster according to equation (S13), provided that $\Delta\theta_{ij} < \theta_C$ and $\Delta\gamma_{ij} < \gamma_C$. At the end of this procedure each remaining unprocessed pixel is removed from the dataset.

The clustered pixels are merged together in a single image in which the different clusters are encoded with different colors. Moreover, for each cluster the pixel color scale intensity represents the integral of the P-SHG spectrum. The cutoff values for γ and θ_F used for performing the clustering analysis of the experimental data are reported in the caption of the corresponding figures.

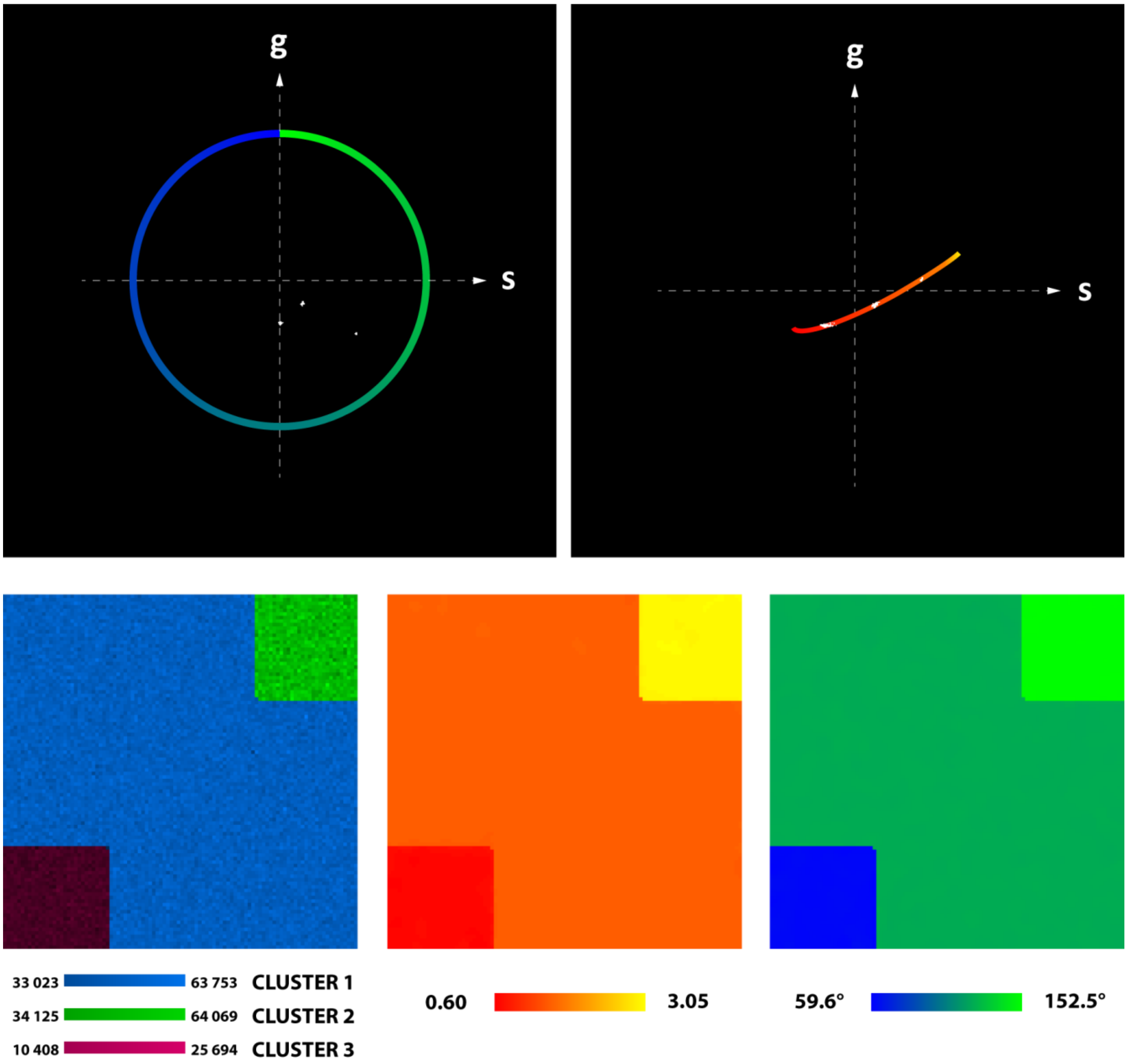

Supplementary Figure 11. Clustering procedure on the θ and γp-plots. Three dataset characterized by different parameters were simulated with the superposition of a Gaussian noise distribution to the SHG signal (standard deviation $5\sqrt{I_{SHG}}$ and mean I_{SHG}). I_{SHG} is given by equation (S8), in which k=1000, y_0=0. Moreover a 3x3 pixels median filter was applied to denoise data. 10000 points were sampled for each simulation. The three simulated set considered are: 1) $\theta_{F1} = 60° \ \gamma_1 = 3$ (30x30 pixels, top right in the image); 2) $\theta_{F2} = 150° \ \gamma_2 = 0.6$ (30x30 pixels, bottom left in the image); 3) $\theta_{F3} = 90° \ \gamma_3 = 1.5$ (the rest of the image). In the upper part of the figure the three dataset are represented in the θ and γp-plots, after the calculation of their Discrete Fourier Transform. In the lower panels the results of the μMAPPS analysis and of the clustering procedure are reported. Left: The image exhibits the obtained clusters merged together. In this case, three clusters were retrieved and encoded with different colors, as shown in the legend. The color scale intensity (reported in the legend) for each pixel of a single cluster represents the sum of its values calculated from the images acquired at different θ_L. The clusters have been obtained with the following cutoff parameters: θ_C=5° and γ_C=0.5. Middle: the image illustrates the color-coded values (as shown in the legend) of the anisotropy parameter γ in each pixel, retrieved from the γp-plot. Right: the image represents the color-coded values (as shown in the legend) of the angle θ_F in each pixel, retrieved from the θp-plot. We remark that the method is able to extract the correct θ_F and γ parameters also if the points lie within the indetermination zone (as point of the dataset 1 and 2).

Supplementary Note 6

Mouse-tail tendon

To further validate our method, μMAPPS has been exploited to analyze polarization-dependent SHG image stack of a mouse-tail tendon, digested by means of collagenase (5mg/ml for 1h at 37 °C). Images of both normal and denatured mouse-tail tendon have been acquired as a function of the laser polarization θ_L within the range [0,2π] with the polarization rotation step $\Delta\theta = 10°$. Supplementary Fig. 12 a and e show the maximum intensity projection of the mouse-tail tendon images acquired as a function of θ_L. The image stack corresponding to the collagenase-digested mouse-tail tendon has been acquired with double the power with respect to the normal control sample due to a reduced emitted SHG signal. By comparing Supplementary Figs. 12a and 12e, a decrease of SHG intensity can be noted throughout the sample together with alternated bright and almost dark areas. Also for thermal denaturation, the formation of tiger-tail like band pattern perpendicular to the collagen fibers, with horizontally extended areas characterized by a reduced SHG signal, has been reported.

In Supplementary Figs. 12b-c and 12f-g the color-coded pixel by pixel θ_F (green-blue) and γ (red-yellow) maps are reported for normal and denatured tendon respectively. The values of θ_F and γ has been extracted as explained in the Main text for Fig. 3. Moreover, Supplementary Figs. 12d and 12h show the normalized clusters obtained with the cutoff parameters $\theta_C = 30°$ and $\gamma = 1$, encoded with different colors, whose scale intensity for each pixel represent the integral of the P-SHG spectrum. The analysis of the normal tendon recognizes 5 different clusters while in the denatured case the number of clusters was 4.

In this case, only areas of the sample not characterized by crimped features has been selected in order to better highlight eventual differences in the anisotropy values distribution. As shown in the histogram of Supplementary Fig. 12i, mouse-tail tendon digested by collagenase did not exhibit a mean anisotropy γ parameters significantly different from the normal control sample, in agreement with the literature ($\bar{\gamma} = 1.53 \pm 0.23$ for normal tendon and $\bar{\gamma} = 1.56 \pm 0.16$ in case of denaturation).

In conclusion, during the denaturation process the intensity of the SHG signal in the mouse-tail tendon is reduced, while the value of the anisotropy value is almost constant, in agreement with the literature[57]. This is probably due to the fact that areas with a compromised microscopic collagen structure are not anymore able to emit an SHG signal and therefore their polarization dependence can no more be tested.

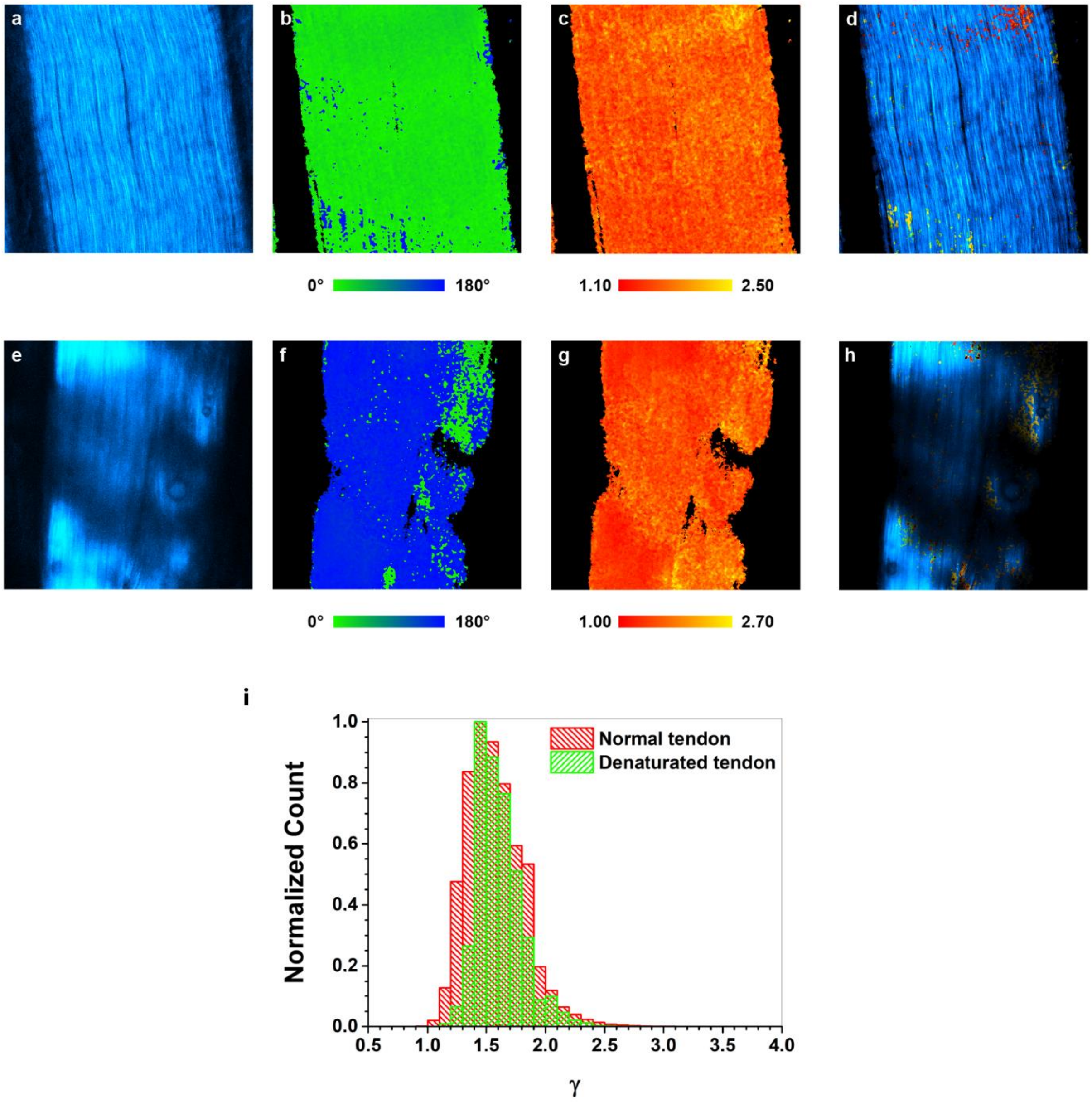

Supplementary Figure 12. Normal and collagenase-digested mouse-tail tendon samples analyzed by μMAPPS a) A stack of images is acquired as a function of the laser polarization θ_L for the normal mouse-tail tendon. In the image the maximum intensity projection of the acquired stack is shown. Image size: 225x225 μm^2. P_{exc} = 10mW (measured before the scanning-head) b) and c) represent respectively the pixel by pixel θ- and γ-maps, where the color codes are shown in the legends. d) 5clusters obtained with the cutoff values θ_C=30° and γ_C=1. Clusters with a number of elements with respect to the total analyzed pixels (ET) > 1% are reported. A main cluster, containing 198˙459 elements, is obtained and shown in blue color. Each color represents a different cluster, while the color scale intensity for each pixel of a single cluster represent the sum of its values calculated from the images acquired at different θ_L. e) maximum intensity projection of the image stack acquired as a function of the laser polarization θ_L for the denatured (collagen-digested, 5 mg/ml for 1 h at 37 °C) mouse tail tendon. Image size: 225x225μm^2. Following the denaturation process, the collagen of the tendon shows an overall reduced SHG intensity with some areas in which no signal or few counts are detected. As a consequence, an increase of laser power P_{exc} = 20 mW (measured before the scanning-head) was necessary to obtain a signal similar to that in a); f) and g) represent respectively the pixel by pixel θ- and γ- maps for the denatured tendon, where the color codes are shown in the legends. h) 4 clusters obtained with the cutoff values θ_C=30° and γ_C=1 for the denatured tendon. Clusters with a number of elements with respect to the total analyzed pixels (ET) > 1% are reported. Also in this case, a main cluster, containing 129˙067 elements, is obtained and shown in blue color. i) Normalized counts histograms of the γ

values obtained for the normal (red) and denatured (green) tendon. The mean γ value for the normal tendon is γ=1.53±0.23, while in case of denaturation the value is slight γ=1.56±0.16. During the denaturation process the intensity of the SHG signal in the mouse-tail tendon is reduced, while the value of the anisotropy value is almost constant, in agreement with the literature. Also the mean angle distribution (not shown) within the image remains almost unaffected by the denaturation process, showing only a slight larger distribution. This is probably due to the fact that areas with a compromised microscopic collagen structure are not anymore able to emit an SHG signal.

We remind that the blue color in the θ-map represents 180° and the green color stands for 0°: they therefore highlight areas in the image with collagen fibrils lying along the same direction. For each analysis T_{NL} = 250.

References.

61. Shen, Y. R. The principles of nonlinear optics, John Wiley and Sons, Hoboken, NJ (2002)
62. Boyd, R.W. Nonlinear optics, Academic Press, Boston (1992)
63. Freund, I., Deutsch, M. & Sprecher, A. Connective tissue polarity. Optical second-harmonic microscopy, crossed-beam summation, and small-angle scattering in rat-tail tendon. *Biophys. J.***50,** 693–712 (1986)
64. Erikson, A., Ortegren, J., Hompland, T., de Lange Davies, C. & Lindgren, M. Quantification of the second-order nonlinear susceptibility of collagen I using a laser scanning microscope. *J Biomed Opt.***12,** 044002 (2007)
65. Kleinmann, DA. Nonlinear dielectric polarization in optical media. *Phys Rev.***126,** 1977–1979 (1962)
